# Visualizations during arithmetic tasks hamper performance and increase cognitive load in 9-year-olds: an fNIRS study

**DOI:** 10.3389/fpsyg.2026.1722948

**Published:** 2026-02-11

**Authors:** Simon Skau, Jimmy Karlsson, Jakob Åsberg Johnels, Ola Helenius

**Affiliations:** 1 Department of Mathematics and Computer Science, Karlstad University, Karlstad, Sweden; 2 Faculty of Education, Department of Education and Special Education, University of Gothenburg, Gothenburg, Sweden; 3 Faculty of Education, Department of Pedagogical, Curricular and Professional Studies, University of Gothenburg, Gothenburg, Sweden

**Keywords:** arithmetic, cognitive load, fNIRS, increase working memory load, pictorial problem, text-based, visual aid, word problem

## Abstract

This study investigates whether visual aids increase or decrease cognitive load during arithmetic tasks in 9-year-old children, using behavioral measures and functional near-infrared spectroscopy (fNIRS). Previous research suggests mixed outcomes regarding whether visual aids increase or reduce cognitive load in educational tasks. In this experiment, children (*N* = 81) completed arithmetic tasks under varying conditions: text-based tasks, visual aid tasks, and irrelevant visual information tasks, all under low and high cognitive load and controlled for arithmetic content. Contrary to our preregistered hypothesis, tasks with visual aids resulted in more errors, longer time to answer, and increased functional activity in the prefrontal cortex (PFC) compared to only text-based tasks, suggesting that visual aids increased cognitive load. Scores on tasks with irrelevant visual information were equivalent to text-based tasks, but time to answer was slower. Implications for future research on cognitive load and theories about cognitive load are discussed, especially what to control for and the distinction between intrinsic and extraneous load.

## 1 Introduction

When children engage with arithmetic tasks such as addition and subtraction, there is a limited number of actual arithmetic tasks they can do, e.g., there are only 45 possible ways to create additive tasks of the type a+b with numbers between 1 and 10. However, even though the task 6+4 and “If Arthur has six flowers and Bruce has four, how many do they have together?” involve the same arithmetic calculation, there is an infinite set of permutations of the latter task. In this pool of infinite possible tasks with the same arithmetic core (e.g., 6+4 ), can we describe different ways in which some are better or worse? For example, one typical difference between arithmetic tasks presented to children is whether they are only text-based word problems, like in the example above, or if they have visual aids (like a picture of Arthur holding six flowers and Bruce holding four flowers), also called pictorial problems. There are many different reasons why one might want to utilize text-based arithmetic tasks or arithmetic tasks with visual aids. If one has the desideratum *the more images the better* or *translate images to arithmetic*, then tasks with visual aids are preferable. But say that we have the desideratum *minimize the cognitive load so that the solver can focus on the arithmetic*; then the task version with the lowest cognitive load is the preferred one. However, some studies give us reason to believe that visualizations increase cognitive load during a mathematical task (van Lieshout and Xenidou-Dervou, 2018; Berends and van Lieshout, 2009; van Lieshout and Xenidou-Dervou, 2020), whereas other studies suggest that it reduces cognitive load (Hoogland et al., 2018; Schoenherr et al., 2024). The aim of this paper is to investigate whether tasks with visual aids increase or decrease cognitive load compared to only text-based tasks, based on different types of behavioral measures and functional activity with functional near-infrared spectroscopy (fNIRS).

### 1.1 Cognitive load

Load, as in perceptual or cognitive load or some other load, refers to the amount of information a person is processing relative to their ability to process that information. So why talk about “load” instead of “information being processed”? One reason is that the concept of “load” allows us to discuss the phenomenon of overload, that is, when there is too much information to process to achieve our goals, whether it’s learning something or performing a task. Overload is hence associated with our perceptual or cognitive capacities having certain limitations; i.e., overload occurs when the demands of the task exceed the available resources. With load terminology, “high load” is equivalent to “a large amount of information being processed,” and “low load” means “a small amount of information is being processed.” Information processed can be under our control and attention, called top-down processing, or the processing can be driven by external stimuli that capture our attention, called bottom-up processing. When performing a cognitive task, such as solving a new arithmetic problem, we need to allocate attentional resources in a top-down manner to process the relevant information to succeed with the task. These top-down processes are usually referred to as executive functions or cognitive control (Diamond, 2013). The standard model of executive function suggests three core components: working memory, inhibition, and cognitive flexibility, which differentiate throughout development (Diamond, 2013). Together, these core functions facilitate the higher-level executive functions, such as *reasoning*, *problem solving*, and *planning*. Working memory is the ability to actively manipulate information no longer perceptually present (Diamond, 2013). Inhibition is the capacity to control or prioritize behavior, attention, emotions, and thoughts in a goal-directed manner, whereas cognitive flexibility is the ability to shift attention between different tasks or goals and to switch between different mental sets or perspectives. When a child is trying to solve a new arithmetic problem, working memory provides the workspace where the top-down processing occurs, inhibition helps filter out irrelevant information or prevent it from entering attention, and cognitive flexibility is needed to shift attention between different parts of the problem or change solution strategies. Recent meta-analyses have provided support for this model, showing that mathematical ability correlates with executive function [*r* = 0.365, 95% *CI* (0.304, 0.422)] (Cortés Pascual et al., 2019), with working memory [*r* = 0.35, 95% *CI* (0.32, 0.37)] (Peng et al., 2016), with inhibition [*r* = 0.19, 95% *CI* (0.14, 0.24)] (Zhu et al., 2024), and with cognitive flexibility [*r* = 0.35, 95% CI (0.34, 0.37)] (Santana et al., 2022).

There are several theories that aim to explain and describe cognitive load, with perceptual load theory (Murphy et al., 2016), cognitive load theory (Kalyuga and Plass, 2025), and multiple resource theory (Wickens, 2024) being three of the more prominent examples. In the present work, we draw on perceptual load theory and cognitive load theory, together with the standard model of executive function, to generate our hypotheses and guide the experimental task and study design. Both perceptual load theory and cognitive load theory conceptualize cognitive load as the amount of information being processed in working memory.

Perceptual load theory focuses on the nature of selective attention, particularly whether attention selection operates at an early or late stage of processing, and how different levels of perceptual and cognitive load impact these processes (Murphy et al., 2016). The theory suggests that perception operates within a limited capacity, and when this capacity is filled due to high perceptual load, distractors are effectively ignored at an early processing stage and do not interfere with later stages. Conversely, when the task involves low perceptual load, all available stimuli—including irrelevant ones—are processed, which requires cognitive attention to be allocated at a later stage to filter out distractors through inhibition. This allocation of attention at later stages increases cognitive load, leading to more errors and longer response times on cognitive tasks (Murphy et al., 2016). The perceptual load theory also proposes that there is an inverse relationship between perceptual and cognitive load when it comes to sustaining attention. Thus, it is not only that the lower the perceptual load (e.g., less visual information presented), the easier individuals get distracted; it is also the case that the higher the cognitive load (e.g., the more information that needs to be kept in mind and manipulated in working memory), the easier individuals get distracted as well (Brockhoff et al., 2022; Matias et al., 2022).

The cognitive load theory (Sweller et al., 2019) focuses on how to design instruction and defines load as determined by its element interactivity (i.e., elements that need to be processed simultaneously) of the task (Sweller, 2010). Within cognitive load theory, there are two types of cognitive load; *intrinsic* (the inherent complexity of a task^1^), and *extraneous* (cognitive load that is imposed by the instructional design). The theory describes that these two forms of cognitive load are additive (Sweller et al., 2019). Thus, if we want to increase the chances that someone learns something or solves a task, cognitive load theory prescribes that we should focus on reducing the extraneous load as much as possible through easier instructions and tasks that one is equipped to solve with existing knowledge. The intrinsic load is also dependent on what is called *germane* processes, such as the individual’s prior knowledge and knowledge schemas. That is, the same task could have different intrinsic loads for different individuals if their knowledge schemas are different (Sweller et al., 2019; de Jong, 2010).

Building on the integration of executive function, perceptual load theory, and cognitive load theory, we propose that tasks with visualizations reduce cognitive load by optimizing the allocation of cognitive resources through the externalization of task-relevant information, offloading cognitive processes onto perceptual processes (Hegarty, 2011), thus decreasing the burden on working memory. Conversely, the lack of visual information forces the individual to retain all information in mind while trying to extract the relevant details and perform cognitive manipulations within working memory. This increases the risk of distraction and loss of focus, requiring the allocation of additional cognitive resources, such as inhibition and cognitive flexibility, to concentrate on the task-relevant information, which ultimately increases cognitive load.

Related to the increase/decrease of cognitive load by visual aid, cognitive load theory researchers have found different cognitive load effects^2^, i.e., empirical regularities about what changes the learning/performance. Several of these effects could potentially complicate the hypothesized association between visualizations and reduced cognitive load during arithmetic processing. Of relevance are the split attention effect, seductive details effect, the modality effect, redundancy effect, and expertise reversal effect. The *split attention effect* occurs when a person must mentally integrate two or more sources of information (e.g., a diagram and separate text, such as the legend), imposing a heavy extraneous load on working memory. Spatial contiguity is when physically integrating these sources (for instance, embedding labels directly into the diagram) reduces the load and leads to better performance/learning (Ozcelik, 2025). Temporal contiguity is when integrating sources of information in time reduces the load and leads to better performance/learning (Ginns, 2006). *Seductive details effect* is the phenomenon that learning is hampered when interesting but irrelevant information that is not related to the learning goal/instructional objective is presented, compared to when it is removed from the to-be-learned material/instruction (Rey, 2012; Sundararajan and Adesope, 2020). In other words, “irrelevant” here refers to information that does not support solving the task or understanding the target concept. Since learners process the irrelevant information, the extraneous load rises, and fewer resources are allocated to learning the material. The modality *effect* arises under split-attention conditions when one source of information is moved from the visual to the auditory channel (e.g., spoken text alongside a diagram), effectively increasing usable working-memory capacity and enhancing performance/learning—but only for high element-interactivity material that otherwise would overload a single (visual) channel (Sweller et al., 1998). The *redundancy effect* occurs when two or more sources of information each fully convey the same content (e.g., a self-explanatory diagram accompanied by text that merely describes it). Because learners must still process the redundant source, extraneous load rises, and performance suffers; eliminating or separating the redundant material improves outcomes (Sweller et al., 1998). Finally, the *expertise reversal effect* captures the fact that, as learners gain expertise, guidance that once helped can impose extraneous load, i.e., removing the now-redundant aid improves expert performance (Kalyuga et al., 2003).

### 1.2 Brain activity correlates

From studies on mathematical cognition, we know that the functional network of number processing is made up of frontal–parietal structures in children (Arsalidou et al., 2018), where the posterior parietal cortex (PPC), and particularly the inferior parietal sulcus, has been linked to the development of quantity representation and the interplay between visuospatial abilities and the number line (Nemmi et al., 2016). The same networks, with additional nodes in the prefrontal cortex (PFC), are involved during arithmetic calculations (Arsalidou et al., 2018). The involvement of more frontal areas, such as the frontal polar area (FPA), the dorsolateral prefrontal cortex (DLPFC), and the anterior cingulate cortex, increases when there is an increased demand on working memory or when goal maintenance through cognitive flexibility or inhibition is required (Menon, 2015). In studies with 9- and 10-year-olds (Obersteiner et al., 2010; Dresler et al., 2009) and adults (Richter et al., 2009), it has been shown that text-based tasks increase the activity of the PPC. In a study by Iuculano et al., it was shown that children aged 7–9 with mathematical learning disabilities had increased and more widespread functional activity in both frontal and parietal areas during mathematical tasks compared to typically developing children, but that this increased activity “normalized” after 8 weeks of one-on-one mathematical tutoring (Iuculano et al., 2015). This is in line with Soltanlou et al. (2018b), who, in their study with typically developing children aged 10–11, showed that with increased performance on arithmetic tasks after a 2-week intervention, they also found a decrease in activity in the left angular gyrus and right middle frontal gyrus. Thus, there is reason to believe that at the stage of mathematical proficiency (and/or development) that primary school children are in, they rely to a large extent on domain-general cognitive processing when solving arithmetic tasks (Soltanlou et al., 2017). However, with increased proficiency and less reliance on working memory and executive functions, the involvement of the PFC decreases and that of the PPC increases (Peters and De Smedt, 2018; Soltanlou et al., 2018a).

Based on what we presented in the previous section, a change in cognitive load is taken to be synonymous with a change in executive function, and in particular, working memory. However, most studies of cognitive load within educational psychology settings do not evaluate cognitive load by linking it to objective measures of working memory but rather through subjective self-report (Krieglstein et al., 2023). The problem is that executive functions and working memory are theoretical constructs; thus, change in working memory not related to a specific working memory task has been hard to evaluate. However, within cognitive neuroscience, there is a large body of evidence indicating that the prefrontal cortex is part of the neurocorrelate of executive functions and working memory (Breukelaar et al., 2017). Thus, by measuring functional brain activity in the frontal cortex, it would be possible to capture changes in cognitive load. Importantly, however, changes in activity patterns could be related to changes in intrinsic (e.g., task complexity) or extraneous load (i.e., distractions); thus, experimental control is needed for activity patterns to inform our models of numerical cognition. Although not much direct prior research has been done, there are some studies showing promising results^3^. For example, in a fNIRS study by Soltanlou et al. (2017), they showed increased functional activity in several areas of the frontal–parietal network when the intrinsic difficulty increased (from one-to-two-digit multiplication). Moreover, in a recent fMRI study, Ozcelik showed that separating the labels of the bars in a graph from the legend increased the extraneous cognitive load as well as increased activity not only in the visual areas but also in the frontal–parietal network (Ozcelik, 2025). The aspects described above generates some conceptual and methodological challenges for our purposes. We know that

- The frontal cortex is involved in solving arithmetic tasks for children,
- Increased use of working memory involves increased activity in the frontal cortex.
- Task-relevant difficulty (intrinsic load) and task-irrelevant difficulty (extraneous load) are both understood as increasing the involvement of working memory.

**Figure 1:**
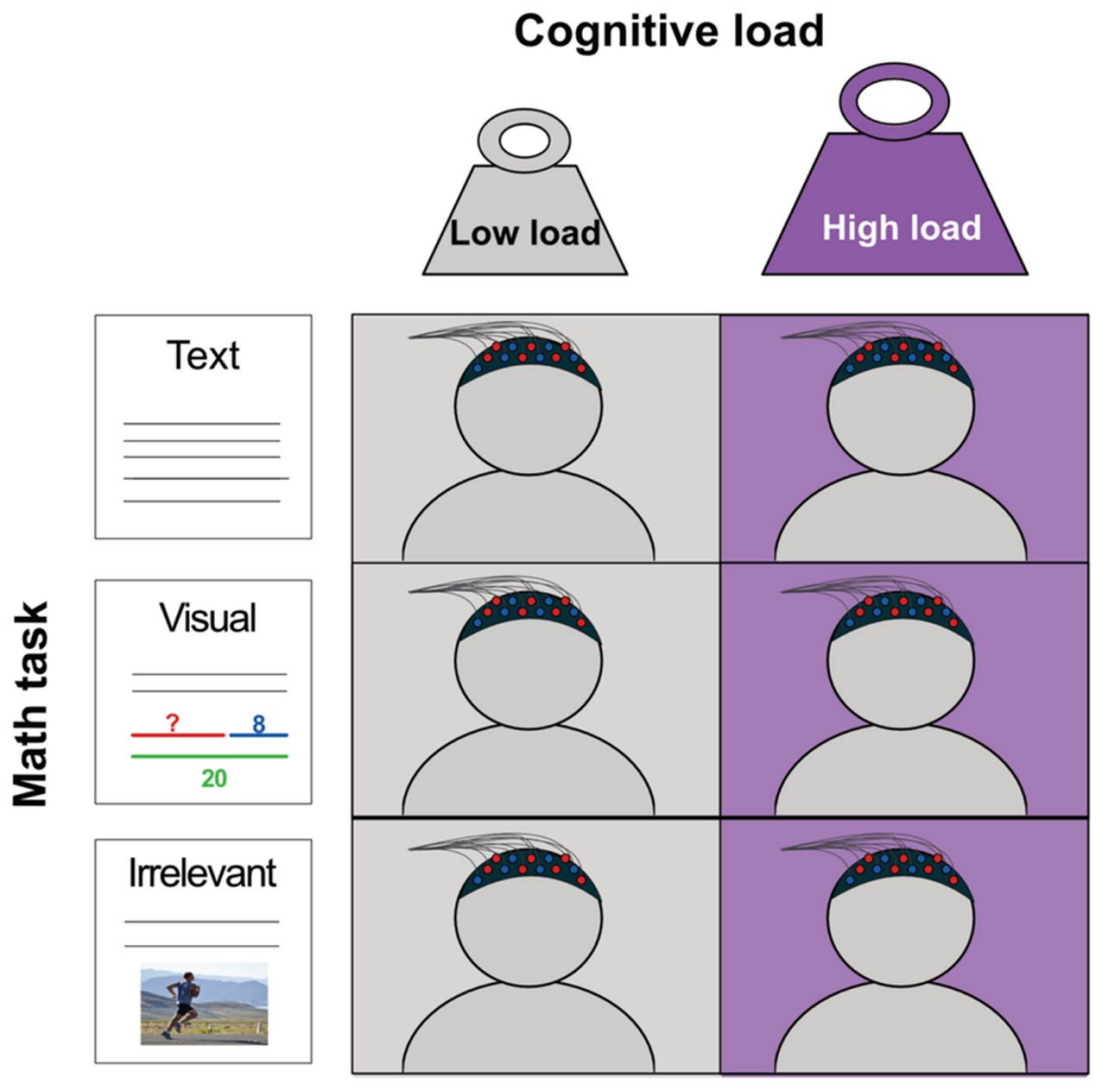
Presentation of the experimental design and the increase working memory paradigm. There are three types of tasks (text-based, visual aid, irrelevant) and two loads (low, high).

The problem that can arise from I, II, and III is that when we use the functional activity of the PFC as an indicator of cognitive load, we cannot separate “task A is more difficult than task B” from “tasks A and B are equally difficult, but task A involves working memory more.” To handle this problem, we have, in the current study, invented a design where we not only match the intrinsic load between tasks with relevant visual aid, irrelevant visual aid, and text-based formats, but also added conditions where we increase the cognitive load by enhancing the involvement of working memory. This is achieved by requiring participants to keep information in mind between tasks, which we call an increase working memory paradigm (Figure 1). With this *increase working memory paradigm*, we can identify which areas are involved only for an increase in working memory and specify our analysis in these areas to compare the cognitive load between the different tasks. This experimental paradigm has the potential to isolate increases in working memory during the task to measure cognitive load that does not rely on subjective measures. It does not rely on the use of a working memory task such as N-back or digit span as a covariate or the use of a dual-task design that involves two types of performance and cognitive flexibility. Ayres et al. (2021) argued that mathematical tasks exclusively using symbolic representations (e.g., 6+4 ) involve only intrinsic load and no extraneous load. Thus, mathematical tasks with the same arithmetic core but presented differently will only represent a change in extraneous load.

### 1.3 Visual aid and cognitive load in mathematics

There are several studies that give credence to the idea that visual aids lower the extraneous load during mathematical tasks. Hoogland found a small effect size, Cohen’s d of 0.09 for visual aid compared to text-based formats in a study of 31,000 10- to 20-year-olds (Hoogland et al., 2018). In a recent meta-meta-analysis of the cognitive load effect of multimedia (defined as information in both verbal and visual form), it was found that text + visual aid had a positive effect on cognitive load and learning (Hedges’ *g* of 0.62 and 0.39, respectively) (Noetel et al., 2021). Similarly, in a meta-analysis of Richard Mayer’s work on cognitive load in multimedia, similar effects of text + visual aid were found (Hedges’ *g* of 0.45) (Cromley and Chen, 2025). Both studies showed that the removal of seductive details decreased cognitive load (Noetel et al., 2021; Cromley and Chen, 2025). When investigating the effect on age, however, they found that multimedia techniques helped middle school and older children, but not elementary school children (Cromley and Chen, 2025).

In a series of studies, van Lieshout and colleagues have found that presenting mathematical problems in pictorial form reduces performance, which they interpreted as increased cognitive load (van Lieshout and Xenidou-Dervou, 2018; Berends and van Lieshout, 2009; van Lieshout and Xenidou-Dervou, 2020). However, it was only in one study, with middle school children, where the participants read the text-based information themselves; in that task, the symbolic representation of the task was also present (e.g., 4 + 6) (Berends and van Lieshout, 2009), which, according to (Ayres et al., 2021) classification, would not involve any extraneous load. With elementary school children, they found a modality effect (van Lieshout and Xenidou-Dervou, 2018), i.e., increased performance when visual aid was paired with the task read aloud to them; however, this effect was not replicated in (van Lieshout and Xenidou-Dervou, 2020); thus, further research with considerations for confounding variables is clearly needed.

### 1.4 The current study

**Figure 2:**
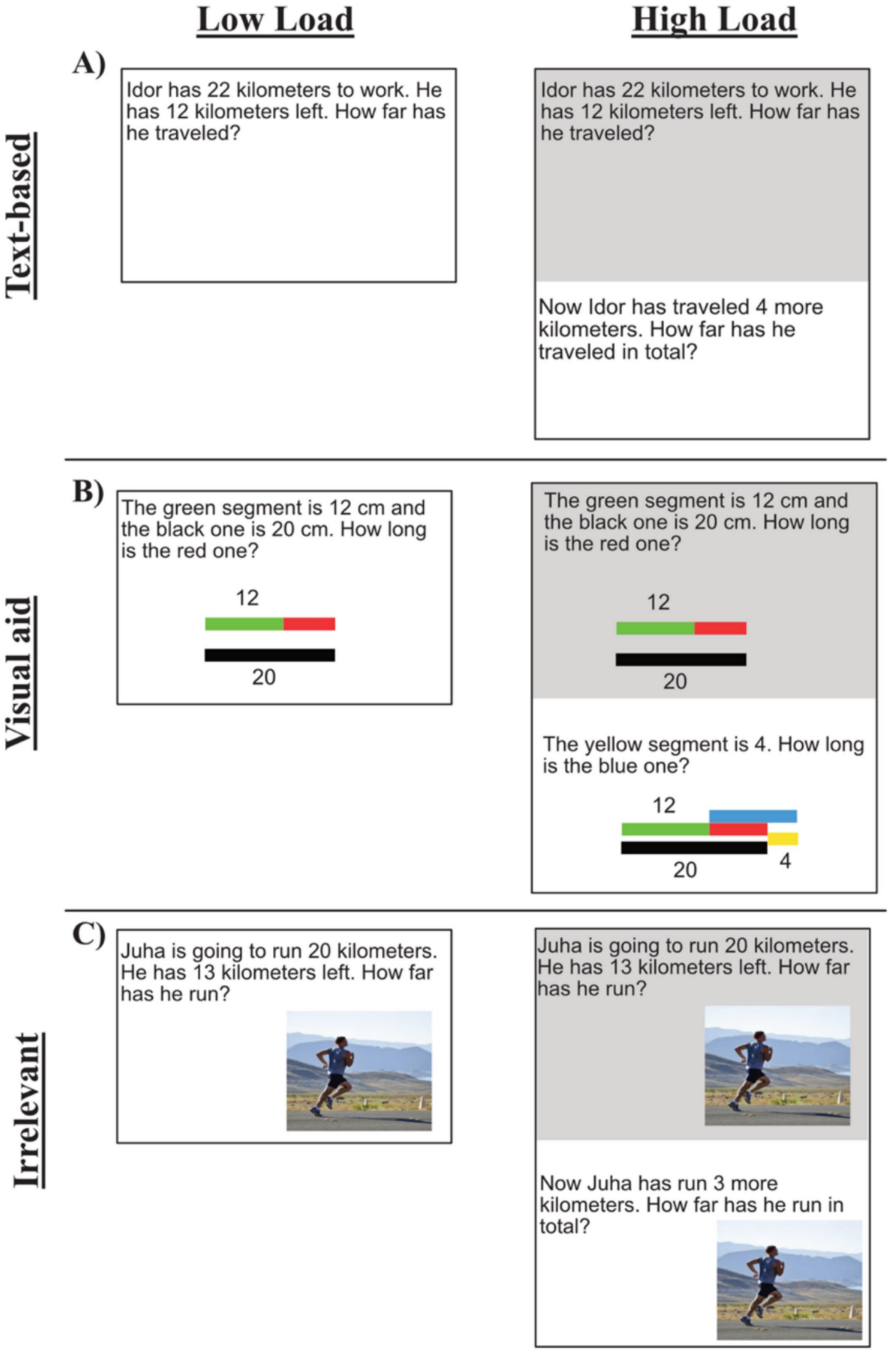
Example of the mathematical task under the different conditions: **(A)** Text-based, **(B)** visual aid, and **(C)** irrelevant condition. The left column is the low load condition and the right column is the high load condition. The grey part is the same as the low load condition and was always presented together with the high load condition. For the other conditions and the arithmetic matchings see https://osf.io/mprcy/files/osfstorage.

In our previous study (Skau et al., 2022a), we also observed some contradictory results between text-based tasks and tasks with visual aid. In that study, as in this, we used fNIRS to measure functional activity. fNIRS is a child-friendly brain imaging technique that uses near-infrared light to measure the relative change in oxygenated and deoxygenated hemoglobin (oxy-Hb/deoxy-Hb) as an indirect measure of neural activity a couple of centimeters down into the cortex (Scholkmann et al., 2014). Both text-based and visual aid tasks generated increased functional activity in the PFC; one channel in the medial PFC indicated greater activation for text-based tasks compared to tasks with visual aid, suggesting a higher cognitive load. However, the children made *more errors* during visual aid tasks (35% vs. 21%), indicating higher difficulty or higher cognitive load compared to the text-based task. Since we did not collect response time data in this study, we were unable to elaborate on the inconsistency. Also, since we only had two conditions and were thereby stuck in the methodological problem generated in I, II, and III, as discussed in the previous section, additional studies were needed to experimentally untangle the difference. In the previous study, all the added visual information was relevant, and we speculated that even if more information is processed, the relevant information decreases the cognitive load; however, since we did not have a condition with irrelevant visual information, this could not be concluded with certainty. Thus, in the current study, as illustrated in Figures 1, 2, the mathematical tasks are created based on task types: visual aid (Visual), text-based (Text), and irrelevant visual aid (Irrelevant) during low and high load conditions. High and low load should here be understood in relative terms, where high load is only denoted high since it is higher than the low load. The high load is higher than the low load since the tasks are matched for intrinsic load, but in the high load condition, one of the terms is the answer from the low load condition. That is, they need to keep the low load information in working memory to solve the high load condition, thus increasing the working memory load (Figures 1, 2).

Our hypotheses are (with the statistical hypotheses in parentheses):

Hypothesis I: More correct answers on visual low load compared to text low load (i.e., BF_10_ > 3 for visual low load compared to text low load scores).

Hypothesis II: Faster time to answers on visual low load compared to text low load (i.e., BF_10_ > 3 that times to answer are faster for visual low load than text low load).

Hypothesis III: No difference in correct answers between text low load and irrelevant low load (i.e., BF_01_ > 3 that there is no difference in scores between text low load and irrelevant low load).

Hypothesis IV: No difference in time to answers between text low load and irrelevant low load (i.e., BF_01_ > 3 that there is no difference in time to answer between text low load and to irrelevant low load).

Hypothesis V: Lower oxy-Hb peak in visual low load compared to text low load (i.e., BF_10_ > 3 that peak oxy-Hb is greater for visual low load than text low load).

Hypothesis VI: Similar oxy-Hb peak in text low load compared to irrelevant low load (i.e., BF_01_ > 3 that there is no difference in oxy-Hb peak between text low load and irrelevant low load).

## 2 Method

### 2.1 Participants

Participants were recruited from third-grade classes in two Swedish primary schools located in a medium-sized city in Sweden. Legal *guardians* (parents or caregivers) gave their written informed consent before the study began, and the participants were told that they could withdraw at any time. In total, 81 children from the two schools participated in the study (31 from one and 50 from the other). The cohort consisted of 39 boys and 42 girls. The data collection was conducted during the months of September and October, in the year they turned 9 years of age. Based on observation of writing, only 3 children were left-handed. The study was approved by the Swedish Ethical Review Authority (Drn: 2024–01002-01).

### 2.2 Experimental design

The study was preregistered at https://osf.io/mb2a8. The study used a within-subject design with one individual test session per child and two whole-class sessions during the same time period. The current paper focuses on the Math task. Data from the other mathematical tasks are included only to support convergent validity and evalute the expertise reversal effect. The math task followed a 3 × 2 within-subjects design as illustrated in Figures 1, 2. Task (3: visual aid, text-based, irrelevant) was one factor, and load (2: low, high) was the other.

### 2.3 Procedure

For the individual session, all tests, including the fNIRS recordings, were performed in a separate room near the children’s classroom. The data were collected with the participant seated in a chair at a table with a computer screen. Before doing the Math task, all participants completed a computerized version of the AX-continuous performance task (AX-CPT), a test measuring proactive and reactive cognitive control (Skau et al., 2021a, 2022a). After the AX-CPT, the fNIRS cap (EASYCAP GmbH, Germany), with the fNIRS optodes and detectors attached, was carefully placed on the participant’s head. Next, the participants performed the Math task. Afterward, they completed a modified version of the Wisconsin Card Sorting Test (WCST), the Additive Situation Task, and Digit Span. The procedure took between 40 to 60 min in total, including preparations and training runs for all tasks.

During the whole-class test, the children performed two additional mathematical tasks: *Basic numeracy and calculations (BANUCA)* (Räsänen, 2005) and *Additive and multiplicative reasoning* (AMR) (Nunes et al., 2010). In the current paper, the main analyses focus on the Math task, and the other tasks are used only to support the convergent validity of the Math task and to check the expertise reversal effect.

### 2.4 Measurements

#### 2.4.1 The math task and the increase in working memory paradigm

The author, O. H., developed the test based on Vergnaud’s theoretical analysis of the psychology of the addition and subtraction operations (Carpenter et al., 1982). An additive situation is any situation that can be modeled by addition or subtraction. For the test, four classes of situations were considered. *Static adding* (You have 2 red marbles and 3 blue, how many do you have together?), *dynamic adding* (You had 2 marbles and won 3, how many do you have now?), *dynamic subtraction* (i.e., take away; You had 5 marbles and lost 3, how many do you have now?), and *comparative subtraction*, (Peter has 5 marbles and Rosa has 3, how many more does Peter have?). Each situation type can be formulated in an open form, such as you had 2 marbles and won some and now you have 5, how many did you win? This can formally be modeled by the arithmetic expression 2+_=5 but can also be calculated as 5−2=_ . In a similar style, the three other classes of situations all have corresponding open formulations, forming a total of 8 possibilities. The test contained 60 arithmetical tasks based on the 8 additive situation types described above.

In the *increase working memory paradigm*, all tasks come in pairs n and n’, with the low load task n being followed by a high load task n’ (see Figure 2). The n’ task was of a similar type as the n task, and the extra load in n’ was created by letting the answer to n serve as a premise for n’, supposedly increasing working memory load. As shown in Figure 2, the n’ tasks include a shaded version of the n tasks. One third of the tasks were formulated in natural language (Swedish), describing an additive situation (text-based) (Figure 2A). One third of the tasks consisted of similar additive tasks, matched for arithmetic difficulty, but were formulated with the help of a picture involving spatial/geometric information, such as length, distance, or movement (geometric support) (Figure 2B). One third of the tasks were similar to the text-based tasks but included a picture with irrelevant information, chosen so it would neither be required nor helpful (text-based irrelevant) (Figure 2C). In the visual aid tasks, the pictorially communicated situation was required for solving the task.

The three types of tasks were randomly mixed so that the order of the tasks was not all visual aid, then all text-based, then all irrelevant tasks. All participants performed the tasks in the same order. For each task, participants were handed a sheet of paper describing/depicting the task. The researcher read the task aloud to the participant twice with a short pause in between. Participants had 30 s to write down the answer before the sheet of paper was removed. Four to six seconds later, the next sheet of paper with a task was presented. The participants were informed that they could write on the sheet of paper if they wanted to make sketches or calculations. The raw scores were defined by the number of correct answers and the time taken to answer. If it took more than 30 s to answer, the response was considered incorrect, and the time variable was capped at 30 s. This was because, in some cases, children took longer than 30 s to respond. The researchers decided it was better in some cases to allow the child to continue answering until they were satisfied, as this approach helped keep the children engaged and motivated. These decisions were made to ensure the participants’ comfort and motivation during the task. The tasks and property comparison of the tasks can be found at https://osf.io/mprcy/files/osfstorage. The low load text based and low load visual aid are the same as in Skau et al. (2022a).

#### 2.4.2 Additional mathematical tests

*Additive situations* (Skau et al., 2022a). The author, O. H., developed the test with the same principals as described in the previous section. The children were asked to listen to a description of an additive situation involving two numbers. They were then asked to choose the arithmetic expressions that described the situation out of four possible choices presented on the computer screen. The arithmetic expressions were of the form a+b=_,a−b=_,a+_=c or a−_=c with only one correctly modeling the situation. The children did not need to carry out any calculations, only pair the situation to the correct expression. Each arithmetic expression was highlighted with a specific color and presented at different sites on the screen. The arrow keys changed to have four different colors. The participants responded by pressing a colored button on a keyboard corresponding to the specific color of the arithmetic expression, which was located on the same side of the keyboard as on the screen. There were a total of 17 trials, of which 7 used numbers below 10 and the rest used numbers in the 20–100 range. Each question was verbally presented and, within a few seconds, repeated once. The participant had 25 s to answer before the trial ended, and there were 4 to 6 s between the end of a trial and the beginning of the next. All participants performed the trials in the same order. The task was done individually during the experimental session. The number of correct answers was recorded as a raw score.

*Basic numeracy and calculations (BANUCA)* (Räsänen, 2005). The test assessed the basic non-symbolic and symbolic number sense, subitizing and conceptual subitizing, simple addition and subtraction, and the ability to identify simple number patterns. We only used the calculation parts; additionally, we added ten simple multiplication questions. The test was administered to the whole class. We used the total score (maximum score of 65 points) as the outcome.

*Additive and multiplicative reasoning* (AMR) (Nunes et al., 2010). The test contained a total of 22 tasks. About half of the tasks required additive reasoning, such as “Jamal and Sara play a game. Sara is on number 11 and Jamal on number 4. How much further ahead is Sara?” (accompanied by a picture of the board game with Sara’s and Jamal’s positions shown). Half of the tasks were multiplicative, such as “There are 3 rabbits in each house, how many rabbits in total live in the 4 houses?” (accompanied by a picture of 4 houses with the digit 3 on one of them). The test was administered to the whole class. We used the total score (maximum score of 22 points) as the outcome.

### 2.5 fNIRS data acquisition

The fNIRS measurements were performed using a continuous wave system (NTS, Optical Imaging System, Gowerlabs Ltd., UK) (Everdell et al., 2005) with two wavelengths (780 and 850 nm) to measure changes in the concentration of oxygenated hemoglobin (oxy-Hb) and deoxygenated hemoglobin (deoxy-Hb). The system has 16 dual-wavelength sources and 16 detectors. The array is the same as we have used in previous studies (Skau et al., 2022b) and is made up of 44 channels (i.e., source/detector pairs) with a source–detector distance of 30 mm and two short-separation channels with a 10-mm distance, as suggested by previous studies (Gagnon et al., 2011; Brigadoi and Cooper, 2015). Short-separation channels are only sensitive to hemodynamic activity in the scalp and skull. Since the regular channels measure signals originating in the brain and the scalp and skull, the use of short-separation channels allows regressing out the scalp signal to improve the specificity of the fNIRS measurement for hemodynamic responses from within the brain (Gagnon et al., 2011; Brigadoi and Cooper, 2015). The optode placements were designed to encompass the whole of the frontal cortex. The fNIRS data were acquired at a sampling frequency of 10 Hz. Preparation of the fNIRS recording, including placing the cap on the head and removing hair to optimize signal quality, took between a few seconds and a couple of minutes. fNIRS recordings were only done during the Math task and Additive Situations and were kept on but with no recording during WCST. During the Math task, markers were manually added to the recording by the researcher when each task was presented.

### 2.6 fNIRS data analysis

The fNIRS data were preprocessed using MATLAB 2018b (MathWorks Inc, 2018) and the MATLAB-based fNIRS-processing package Homer3 (Huppert et al., 2009) (pipeline can be found at https://github.com/SimonSkau/NCM_study/blob/main/preprocessing_fnirs). The processing pipeline started with pruning channels using the HomER3 function *hmrR_PruneChannels*, where channels were rejected if their mean intensity was below the instrument’s noise floor (1e-4 AU) and the standard deviation of the signal-to-noise ratio was above 1. Raw data were then converted to optical densities. A high band-pass filter of 0.01 Hz was used to correct for drift and a low band-pass filter of 0.5 Hz to remove pulse and respiration artifacts. The functions hmrR_MotionArtifactByChannel and *hmrR_MotionCorrectSpline* with the recommended value of 0.99 were used to correct for motion artifacts. The function *hmrOD2Conc* was used to convert optical density to hemoglobin concentration with partial pathlength factors of [5.6 and 4.6] set according to age based on (Scholkmann and Wolf, 2013). To calculate the hemodynamic response function (HRF), the *hmrR_GLM* function in HomER3 was used, which estimates the HRF by applying a general linear model (GLM). To solve the GLM, an iterative weighted least-squares fit of a convolution model was applied, in which the HRF at each channel and chromophore was modeled as a series of Gaussian basis functions, with a spacing and standard deviation of 0.5 s (Ye et al., 2009). The model did not include any polynomial drift regressors due to the use of a high band-pass filter. The regression time length was −2 to 25 s. The choice of regression time length was based on the time to answer data, and 30 s was deemed too long, as it could involve too much data from other trials. Due to the wavelength combination of the NTS-fNIRS system (780 nm, 850 nm), oxy-Hb is typically estimated with higher sensitivity than deoxy-Hb (Sato et al., 2013; Uludağ et al., 2004). Thus, oxy-Hb was used as the primary outcome, while deoxy-Hb was analyzed in parallel as a quality check using the same GLM framework, regression time window, and peak-based feature extraction.

### 2.7 Statistical analysis

We chose a Bayes factor (BF) analysis using the open-source program JASP version 0.19 (Marsman and Wagenmakers, 2017). We have applied BF_10_ as the main criterion, and the interpretation of BF_10_ = 3 would be that, given the data, the alternative hypothesis (H_1_) is 3 times more likely than the null hypothesis (H_0_), while BF_10_ = 0.3 can be interpreted as that, given the data, H_0_ is 3 times more likely than H_1_. This can also be expressed as BF_01_ = 3. This means that in a Bayes factor analysis, one does not need to conduct both a difference and an equivalence test, since difference and equivalence are evaluated from the same test statistic (Rouder et al., 2009). Simulation studies suggest that a decision criterion of BF_10_ > 3 corresponds approximately to an *α* = 0.01 in the frequentist tradition (Jeon and De Boeck, 2017). H_0_ is defined in this study as no difference or no association, depending on the test. Following the praxis of Wagenmakers and colleagues (Wagenmakers et al., 2011), a BF_10_ in one of the five categories between 1–3, 3–10, 10–30, 30–100, or above 100 is interpreted as *anecdotal, substantial, strong, very strong,* or *extreme* evidence for H_1_, respectively.

Five preliminary analyses on the behavioral data were conducted, primarily so we can trust the functional activity data, but they are also important for the interpretation of the behavioral data. First, a McDonald’s *ω* to evaluate internal consistency with a value over 0.7 indicates good internal consistency. The analysis resulted in an ω of 0.80 with CI [0.73, 0.86] for visual aid, 0.80 with CI [0.74, 0.86] for text-based, and 0.76 with CI [0.68, 0.83] for irrelevant tasks.

A unidimensional Rasch model analysis was conducted to estimate the item difficulty parameters (*β*) for the 60 tasks, to evaluate their difficulty levels (Braeken and Tuerlinckx, 2009). The aim of the Rasch analysis is to get an estimation of item difficulty; if the tasks are overall too hard, it would affect the interpretation of the fNIRS data. The β values indicate the difficulty of each task, with higher values corresponding to more difficult tasks and values under 0 indicating easier tasks. The analysis showed that the test would not be considered hard, as the overall mean and SD are −1.23 (±1.59) (see Supplementary material).

The second preliminary analysis is to evaluate if there are any fatigability effects (Skau et al., 2021b), i.e., are the tasks harder or does the time to answer increase the longer the test goes. If there is a fatigability effect, then combining results from items at the beginning and at the end will be problematic, which will affect not only the fNIRS but also the analysis of the behavioral data. The fatigability effect analysis was conducted by correlation and paired *t*-test. The analysis suggested no reason to believe that there is any fatigability effect; if anything, the children actually performed better on the tasks towards the end compared to the beginning (see Supplementary material).

The third preliminary analysis is to see whether the scores and time to answer correlate. This is done with Kendall’s *τ*, which has a similar interpretation as Spearman’s *ρ*. The correlation between time to answer and score tended to be stronger within each task condition (see Supplementary material).

The fourth analysis is to compare the Math task performance with the other mathematical tasks. To do this, we z-transform the scores and time to answer and take the average of the two as our dependent variable, correlating it with the raw scores from Additive Situation, BANUCA, and ARM. This, in addition to the theoretical and methodological justification presented in 2.3.1, is to see that the Math task also measures relevant mathematical ability. The correlation with the other mathematical tasks provided strong evidence for a medium to strong correlation, with the weakest correlation for irrelevant low load and AS *r* = 0.44 and the strongest for overall high load and BANUCA AS *r* = 0.69 (see Supplementary material).

The six hypotheses consist of two hypotheses each for scorers (accuracy), time to answer (speed), and oxy-Hb, i.e., the difference between visual low load and text low load, and equivalence between Text low load and irrelevant low load. For each outcome—score, time to answer, and oxy-Hb—manipulation checks need to be conducted, i.e., between high load and low load, which is done with a paired *t*-test. If the manipulation is successful, there should be fewer correct scores during high load, a longer time to answer during High Load, and higher peak oxy-Hb during high load compared to low load. More specifically, this is also done for each task type, i.e., visual high load compared to visual low load, and the same for the text and irrelevant tasks.

For the fNIRS analysis, peak oxy-Hb was used as the outcome variable. As a manipulation check, a paired *t*-test is used for each channel between high and low load (visual and text). If a *t*-test in a channel results in a positive t-value and a BF_10_ > 3, we conclude that this channel is involved when cognitive load is increased. To handle non-identical fNIRS cap positions and multiple testing, if more than one spatially neighboring channel has a BF_10_ > 3, they will be grouped together to form a region of interest to measure cognitive load (as described in section 1.2). The region of interest is thus based on a group-level analysis. The following analysis will only be conducted with these regions of interest.

First, a similar manipulation check as with the behavioral data will be performed for high and low load for visual, text, and irrelevant tasks. If the t-values are positive and BF_10_ > 3 in these regions of interest, then Hypotheses V and VI will be evaluated by t-test for the region of interest between visual low load and text low load, as well as text low load and irrelevant low load. To further evaluate the differences between tasks, the different tasks will be compared with each other (including both loads).

In the secondary analysis, we performed a Bayesian Repeated Measures ANOVA in JASP. Here we use BF_Incl_ (Bayes factor for including the factor). We used the effect across matched models, i.e., we compared the effect of having the factor to equivalent models stripped of the effect.

### 2.8 Deviation from preregistration

The fNIRS pipeline was an old pipeline based on Homer 2, while the correct and used one based on Homer 3 can be found at https://github.com/SimonSkau/NCM_study/blob/main/preprocessing_fnirs. No specification about how to evaluate the hypotheses pertaining to the fNIRS was specified, e.g., how the region of interest should be defined, etc. We chose the approach depicted in 2.6.

## 3 Result

### 3.1 Performance scores on the main math test

**Figure 3:**
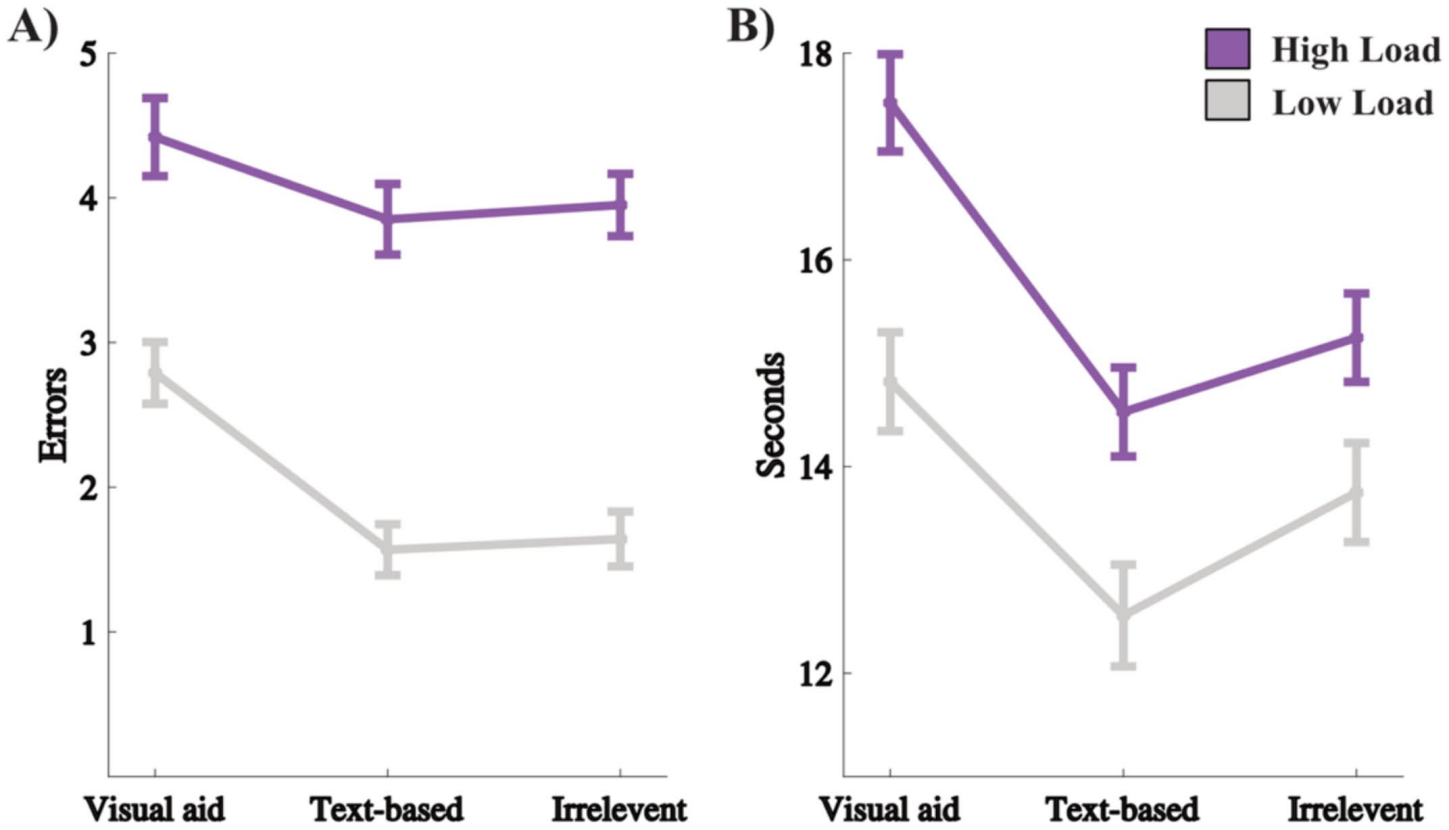
**(A)** Accuracy for each task type and load. **(B)** Time to answer for each task type and load. Error bars are standard error of the mean.

For the analysis of scores (with a Bayesian Wilcoxon signed-rank test), Lower Load resulted in more correct answers compared to Higher Load on an aggregated level with a BF_10_ > 1,000, as well as for each specific task type: Visual low compared to visual high load, text low compared to text high load, and irrelevant low load compared to irrelevant high load (Figure 3A and Supplementary Tables 1, 4). There are fewer correct answers on visual low load compared to text low load (BF_10_ > 1,000) and irrelevant low load (BF_10_ > 1,000), whereas the data supported the null hypothesis (no difference) for text low and irrelevant low BF_10_ = 0.142 (BF_01_ = 6.84).

For the time to answer analysis (with a Bayesian paired *t*-test), the result is similar. Lower Load resulted in faster time to answer compared to Higher Load on an aggregated level with a BF_10_ > 1,000, as well as for each specific task type: visual low compared to visual high load, and text low compared to text high load. For the irrelevant low compared to irrelevant high load, the latter is slower with BF_10_ = 131.05 (Figure 3B and Supplementary Tables 1, 4). Visual low load is slower than text low load (BF_10_ > 1,000) and Irrelevant Low Load (BF_10_ = 35.11). In contrast to the analysis of the accuracy scores, the irrelevant low load task takes longer to answer compared to text low load (BF_10_ = 196.48).

To summarize, the load manipulation seems to work, with the Higher Load condition resulting in more errors and longer time to answer. As behavioral measures, both accuracy and time to answer suggest that the visual low load task results in more errors and takes longer to complete compared to text low load tasks, which is contrary to Hypothesis I and II. Hypothesis III is supported, i.e., no difference in task score between text low load and irrelevant low load. However, Hypothesis IV is not supported, since the irrelevant low load took longer to answer compared to text low load.

### 3.2 fNIRS results

**Figure 4:**
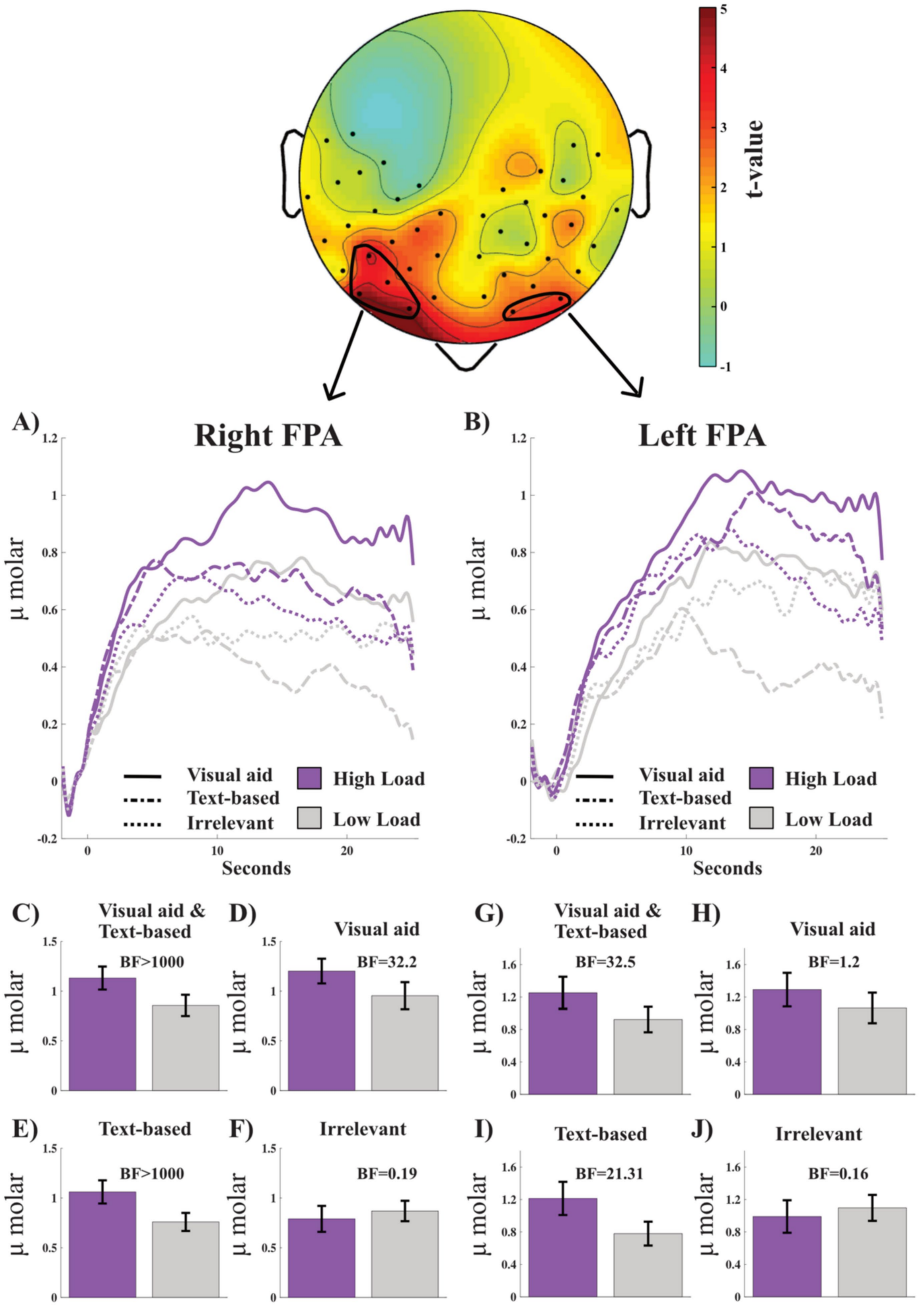
Upper panel shows heatmap based on *t*-values for the difference between oxy-Hb during high load (visual aid and text-based) and low load (visual aid and text-based). See Supplementary Figure 2 for layout of channels. Circled areas are regions of interest where two or more adjacent channels had a BF ≥ 3. **(A,B)** Visualize the oxy-Hb curves for the three conditions and each type of load in the right and left frontal polar (FPA), respectively. **(C–F)** Are bar graphs for peak oxy-Hb in the right FPA, whereas **(G–J)** are for peak oxy-Hb in the left FPA. Bayes factor (BF) values are presented for each comparison in each graph. Error bars are standard error of the mean.

To identify areas involved when cognitive load is increased, a comparison was made between visual and text high load against visual and text low load, respectively. The upper panel of Figure 4 shows the heatmap based on paired *t*-tests for each channel. The circled areas are the channels with a BF_10_ > 3.0 (all test statistics for the heatmap are presented in Supplementary Table 5). Four channels on the right hemisphere were averaged together and denoted as the right FPA, while two channels on the left hemisphere were averaged together and denoted as the left FPA. Figures 4A,B visualize the oxy-Hb curves during the different tasks and loads in the right FPA and left FPA. In the lower panels of Figure 4, bar graphs of peak oxy-Hb in the right and left FPA for the different conditions are presented.

In the right FPA, the high load had a higher oxy-Hb peak compared to low load, with a BF_10_ > 1,000 for visual aid and text-based conditions combined (Figure 4C), for visual aid only BF_10_ = 32.2 (Figure 4D), and for text-based only BF_10_ > 1,000 (Figure 4E), but not for irrelevant conditions, BF_10_ = 0.9 (Figure 4E). For the left FPA, the high load had a higher peak oxy-Hb compared to low load, with a BF_10_ = 32.2 for visual aid and text-based conditions combined (Figure 4F) and for text-based only BF_10_ = 213.5 (Figure 4H). However, the data were inconclusive for a difference in peak oxy-Hb in the left FPA between High and Low conditions for visual aid, BF_10_ = 1.2 (Figure 4G), and showed no difference for irrelevant conditions, BF_10_ = 0.16 (Figure 4I). These results indicate a successful manipulation, and we can continue with the analysis for these conditions and areas. However, since the paired *t*-tests provided evidence for no difference for Irrelevant High compared to Irrelevant Low in both the right and left FPA, we will not proceed with that analysis. That is, we will not be able to evaluate Hypothesis VI, i.e., that the oxy-Hb in text low load and irrelevant low load is equivalent, since we do not trust the fNIRS data of the irrelevant conditions.

**Figure 5:**
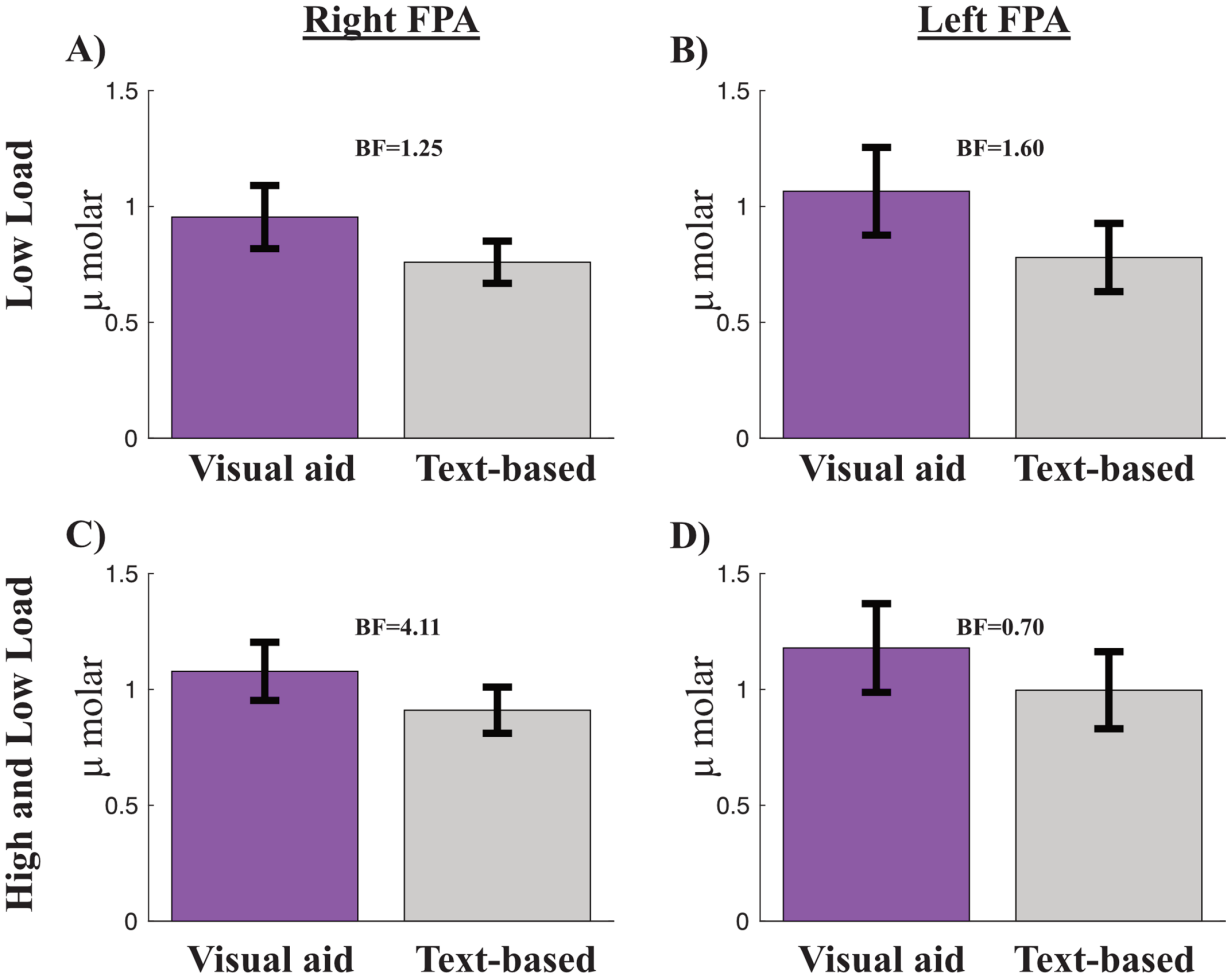
Bar graphs of the comparison between oxy-Hb differences for visual aid and text-based tasks. **(A,B)** are for low load and right and left frontal polar areas (FPA), respectively. **(C,D)** are for both high and low load for right and left FPA, respectively. Bayes factor (BF) is presented in each graph. Error bars are standard error of the mean.

Next, we evaluated Hypothesis V, that text-based tasks will involve higher oxy-Hb compared to tasks with visual aids, as an indicator of higher cognitive load, in the right FPA and left FPA. The paired *t*-test between visual low load and text low load in the right FPA resulted in a BF_10_ = 1.257, i.e., anecdotal evidence for higher activity for the visual task (not text-based tasks) (Figure 5A). Again, the paired *t*-test for the left FPA resulted in BF_10_ = 1.60, i.e., anecdotal evidence for higher activity for the visual task (not text-based tasks) (Figure 5B).

Comparing both high and low visual load to text high and low load in the right FPA resulted in a BF10 = 4.11, indicating substantial evidence for higher oxy-Hb in the right for visual tasks (Figure 5C). The same comparison in the left FPA resulted in a BF10 = 0.70, i.e., anecdotal evidence for no difference (Figure 5D). Thus, contrary to our hypothesis, results showed increased right prefrontal activity in the conditions with visualizations compared to text-based, indicative of increased cognitive load.

Visualization of deoxy-Hb did not show the typical U-shaped curve (Supplementary Figure 4), raising concerns about the interpretability of the deoxy-Hb signal. Therefore, deoxy-Hb was not used for primary inference but was analyzed in parallel as a quality check. Specifically, all contrasts performed for oxy-Hb were repeated for deoxy-Hb using the same GLM framework, regression time window, and peak-based outcome. For all contrasts where oxy-Hb showed evidence for an effect (i.e., BF_10_ > 3 in the expected direction), we verified that deoxy-Hb did not show evidence for a change in the same direction (i.e., not BF_10_ > 3 in the same direction), since concurrent same-direction changes in both chromophores are often indicative of noise rather than underlying neural activity (Kinder et al., 2022). Deoxy-Hb results for the oxy-Hb-defined regions of interest are presented in Supplementary Table 5, and the deoxy-Hb results corresponding to the comparisons done in Figures 4, 5 are presented in Supplementary Table 6. Across these tests, deoxy-Hb did not show same-direction evidence for an effect, which reduces the concern that the reported oxy-Hb effects reflect global/systemic noise.

### 3.3 Secondary analysis

To probe any possible expertise reversal effect, i.e., that the difference between visual- and text-based conditions varies for low- and high-performing participants, we z-transformed the results on AS, BANUCA, and ARM and computed the mean across the three tests. Based on this composite variable, we split the sample into quartiles and compared the top and bottom quartiles (i.e., high and low performers). We performed Bayesian repeated measures ANOVA with task (visual aid, text-based) as a within factor and expertise (high performers, low performers) as a between factor on low load score, low load time to answer, high load score, and high load time to answer. The BF_Incl_ for the interaction between task and expertise was 5.63 for low load score, 0.45 for high load score, 0.37 for low load time to answer, and 0.45 for high load time to answer. The only one suggesting an interaction effect is low load score, but this is driven by a ceiling effect for the high performance (mean of 9.0 for visual aid and 9.5 for text-based, where 10 is max). Thus, we have no reason to believe that there is an expertise reversal effect affecting the conclusions from the experiments described above. See Supplementary Figure 2 for visualization of the interactions.

## 4 Discussion

In the current study, we investigated how visual aid affected cognitive load during a text-based arithmetic task for 9-year-olds in a high ecological environment using behavioral measurements and functional imaging. For the behavioral measures, all manipulation checks cleared, making the evaluation of hypotheses I-IV possible. Contrary to our hypotheses I and II (extreme evidence against them), children made more errors and took longer to answer tasks with visual “aid” compared to only text-based tasks, suggesting an *increase* in cognitive load. Hypothesis III, i.e., no difference in correct answers between Text Low and Irrelevant Low, was supported, with substantial evidence, but contrary to our hypothesis IV (with extreme evidence against it), children took longer to answer tasks with irrelevant visual information compared to text-based. For the functional imaging part, the left and right frontal polar area (FPA) was identified as involved when working with the high load tasks, indicating that these tasks indeed involved working memory more. The manipulation check failed for irrelevant high and low load, and hypothesis VI, comparing text-based tasks with irrelevant visual information, could not be evaluated. Importantly, the manipulation checks did work for the visual and text-based tasks. Again, and contrary to our hypothesis V, there was anecdotal evidence suggesting increased activity in the right FPA during visual aid compared to text-based calculation. When combining both high load and low load tasks, there was substantial evidence that there was increased activity in the right FPA for visual tasks compared to text-based tasks, suggesting increased involvement of working memory and thus cognitive load during visual tasks.

In our previous study, we found that text-based tasks resulted in fewer errors compared to tasks with visual aids, but one channel in the medial PFC showed higher oxy-Hb in that study (Skau et al., 2022a). Since we did not have any third variable, such as time to answer in that study, we could only speculate about the inconsistency between the results. In the current study design, several improvements were made: the addition of the time-to-answer variable, the irrelevant condition, and the increased working memory paradigm, which generated the difference between the low and high load conditions. Together, these made it possible to experimentally isolate the cognitive load difference between visual aid and text-based tasks. We also used a region of interest approach for the oxy-Hb data. These differences could potentially explain deviations from the previous study, since in this study, two or more channels needed to show differences between high and low load to be grouped as a region of interest. The approach taken here does not only handle statistical fluctuations but also makes a clearer comparison between participants, since the fNIRS cap placement cannot be said to be identical for each child. As in our previous study (Skau et al., 2022a), and asked by Soltanlou et al. (2018a), the current study had high ecological validity in that the tasks were performed at the children’s school, with pen and paper, on which they could do calculations if needed, and the tasks were similar to ones they encounter during their mathematical activities.

To repeat, the aim of the current paper was to evaluate whether visual aids increase or decrease cognitive load. The hypotheses and study design were informed by the standard model of executive function, perceptual load theory, and cognitive load theory. The conjectures derived from these theories were that visual aids would reduce cognitive load, which was operationalized into the three hypotheses (I, II, and V): more correct answers, faster response times, and lower oxy-Hb peaks during visual low load compared to text low load tasks. The rationale was that the visual information would increase the perceptual load. This would, in turn, reduce cognitive load in terms of reduced involvement of executive functions such as working memory and inhibition, due to increased resistance to distracting thoughts. It would also reduce cognitive load in terms of allocating information from working memory to the external environment, since the participants would not need to keep the information in mind while solving it. Almost all evidence from this study points to the contrary—which begs the question: why? In the following, we will discuss (i) auxiliary hypotheses and possible confounders such as previously identified cognitive load effects, and (ii) what implications our results have on theories related to cognitive load.

### 4.1 Auxiliary hypotheses and possible confounders

The typical first step when one gets falsifying or unintuitive results is to question some of the auxiliary hypotheses and operationalizations (Meehl, 1990). To start with, what about the so-called cognitive load effects discussed in the introduction? Could they explain these results? As the study was designed, we did not want the result to be affected by reading ability; thus, all questions were read aloud twice. Due to this, if anything, we would assume a benefit during visual aid compared to text-only tasks due to the modality effect, i.e., not overloading one channel (visual or auditory) with information (Sweller et al., 2019; Sweller et al., 1998). Additionally, if anything, we would assume that there would be a redundancy effect during text-based tasks, since the exact same information being read aloud was presented visually (i.e., in text form, so the children could read it), whereas for the visual task, the visual information was different from the instructions; i.e., the redundancy effect would, if anything, generate higher cognitive load for text-based tasks.

Related to the redundancy effect is the expertise reversal effect, i.e., as expertise grows, supporting information increases extraneous load and hinders performance since that information becomes redundant to them (Kalyuga et al., 2003). This was ruled out by the secondary analysis that did not find any interaction between task (visual aid vs. text-based) and expertise (high vs. low performers). Thus, we have no reason to believe that the difference in cognitive load between visual and text-based tasks is due to any expertise reversal effect. We also do not see how a seductive details effect could explain the findings, since the visual aid does not contain irrelevant seductive information, and the task that does contain such information (i.e., the irrelevant tasks) generated higher scores and faster response times (Figure 3).

This leaves the split attention effect, i.e., the decrease in performance when a person must mentally integrate two or more sources of information, be it spatial or temporal (Ginns, 2006). When analyzing the visual tasks with a focus on the split attention effect, two out of the ten visual tasks (task 1.3 and 1.7, presented as task 7 and task 13, see https://osf.io/mprcy/files/osfstorage) force the participant to mentally integrate two or more spatial sources of information. Even though this is the case, the difficulty level of these tasks is not particularly different from the other tasks, with *β* values of −2.1 and −0.43, respectively (Figure 3B and Supplementary Table 6). Although we do not think there are reasons to believe that the cognitive load effects explain the present results, we acknowledge that these effects need to be controlled for more clearly in future studies. Why these tasks were not more difficult is hard to say, but it seems that the element interactivity increase and decrease are not as prominent for elementary school children (Cromley and Chen, 2025).

Other potential confounders, which we have not controlled for in the task design, are (i) that most visual tasks are spatial whereas most of the text-based tasks are about sets (number of objects), and (ii) that some of the visual tasks involved measuring, whereas none of the text-based tasks did so. For the spatial vs. set distinction, it is possible to control for this in future studies; however, it will also affect the range of questions. When the range increases, as in the question 15 + 10, we find it unlikely that a picture of Arthur holding 15 flowers and Bruce holding 10 flowers will help most of the children, thus bringing into question if and how that would be construed as a visual *aid*. On the other hand, a formulation like: *“Two rods have identical lengths of 20 cm. One rod has a 12 cm red section and the rest being green. How long is the green part?”* would represent the same task as Figure 2B but without the need for the figure. However, the price is that the formulation of the question becomes more complex. Perhaps text-based questions with the same geometrical content as a corresponding visually supported task could form more appropriately matched task pairs.

For the second possible confounder, three out of ten tasks (tasks 21, 29, and 43) involved measuring with a ruler. Two of these tasks were indeed harder (*β* values of 0.45 and 0.65 for tasks 29 and 43, whereas task 21 had a β of −2.0; see Supplementary Table 6), which could be due to the additional “reading of” abilities that go beyond pure arithmetical reasoning. Research has shown that the activity of measurement is harder for young children (Reynolds, 2011), especially for tasks where zero is not the starting point (Clements et al., 2022). Reynolds found that children with a good spatial understanding still struggled with standardized measures, such as when a ruler was used (Reynolds, 2011).

One possibility that could explain the results of increased cognitive load in the visualized tasks is that children are more trained at solving text-based arithmetical tasks. That is, perhaps it is much more common for arithmetical problems to be presented like the text-based tasks or to involve irrelevant visual information when taught by their teachers or when working with mathematical tasks during class. This possibility has some support in our data, where the performance in terms of accuracy was equivalent for the text-based and irrelevant tasks. The fact that the participants were slower on irrelevant tasks compared to text-based tasks, but faster than visual aid tasks, could be because the image was noticed during the irrelevant task but not engaged with. This is similar to the results from Brendes and van Lieshout, where irrelevant visual information (which they called useless) did not result in different scores, but slower response times (Berends and van Lieshout, 2009). This slower time to answer could be related to the effect of seductive details (Rey, 2012; Sundararajan and Adesope, 2020; Noetel et al., 2021; Cromley and Chen, 2025). Related to this is the problem of depicting the *dynamic subtraction* in a single snapshot (as pictorial forms are) without simultaneously increasing the difficulty (van Lieshout and Xenidou-Dervou, 2018; van Lieshout and Xenidou-Dervou, 2020).

To sum up the discussion about possible confounders, when analyzing the auxiliary hypothesis and operationalization, there are no obvious problems with the task design that could explain the difference. However, future studies investigating differences between visual and text-based tasks should control for the spatial versus set distinction and avoid adding measurement to the visual aid tasks. Relevant to the discussion of what was not controlled for is what was controlled for. In addition to the increased working memory paradigm, the Math task not only controlled for mathematical content but also involved eight different ways of presenting an additive situation as described in section 2.3.1.

### 4.2 Possible implication for theories about cognitive load

If the results are not explained by problems with the operationalization and task design, then the results suggest lowering the credence of some of these theories or some parts of these theories (standard model of executive function, perceptual load theory, cognitive load theory) (Meehl, 1990). The part related to the standard model of executive function seems to hold up, since the manipulation checks suggested increased involvement of working memory and PFC areas associated with working memory and the other executive functions (Breukelaar et al., 2017).

The relevant part of the perceptual load theory that had bearing on the hypothesis formulation and task design was that there would be a difference in perceptual load between the visual and text-based tasks, and that this difference would make participants more easily distracted during the task with less load (text-based), which would not only result in longer time to answer and more errors but also require additional cognitive resources (e.g., inhibition) to stay focused on the task at hand, resulting in more involvement of the PFC. Since this might be more of an operationalization problem, and we did not utilize any manipulation checks for perceptual load, it is hard to draw any firm conclusions here. One could speculate that the slower time to answer in the irrelevant task compared to the text-based task shows that adding visual information increases the perceptual load. However, we hypothesized that there would not be any difference here. Additionally, recent brain imaging studies on the perceptual load theory have brought into question the inverse relationship between perceptual and cognitive load (Brockhoff et al., 2022), showing that higher cognitive load can have an early filtering-out effect of irrelevant information.

When conceptualized in terms of intrinsic and extraneous load from cognitive load theory, brain imaging studies have found that an increase in intrinsic load also increases the functional activity of the frontoparietal network, in particular the PFC, during both mathematical and non-mathematical tasks (Arsalidou et al., 2018; Soltanlou et al., 2017) and non-mathematical tasks (Ayres et al., 2021). Similarly, studies such as (Ozcelik, 2025) show that adding extraneous load increases the functional activity of the PFC. This is why we used the task design, and due to the successful manipulation check, we have good reason to believe that increased activity in the FPA during the tasks is associated with increased working memory activity, especially when it is accompanied by more errors and longer response times. We also matched the arithmetic tasks to keep the intrinsic load constant (Ayres et al., 2021), so that any increase in working memory, as measured by brain activity, would imply an increase in extraneous load. As mentioned, contrary to our hypothesis, the results suggest that visual “aids” increased the extraneous load. However, this conclusion hinges on one of two assumptions (or both): either (i) the possible separation between intrinsic and extraneous load (for mathematical tasks) (de Jong, 2010), or (ii) that a text-based task and a task with visual aids could have the same intrinsic load. Since both issues relate to how intrinsic load is to be understood, we will discuss them simultaneously.

Intrinsic load is *dependent* on the content of the material in question; i.e., intrinsic load, by necessity, cannot exist or occur unless the content of the material exists or occurs, and the content of the material is not part of the intrinsic load (Mulligan and Smith, 1986). In our case, the content is constant, and as long as that does not change, the intrinsic load should not change unless there is something else that the intrinsic load is dependent on, such as familiarity and other germane processes. If changes in the presentation format affect the intrinsic load, as de Jong documents, some proponents of cognitive load theory have suggested (de Jong, 2010), then the distinction for theoretical and predictive purposes between intrinsic and extraneous load turns out to be useful only in extreme cases, since changing the presentation of the task is the canonical example of how to change extraneous load (Sweller et al., 2019; Sweller et al., 1998).

Other factors that the intrinsic load depends on are the familiarity and other germane processes the individual has with the material in question, which is one of the dynamic parts of the theory that proponents of cognitive load theory bring forth as one of the theory’s desirable characteristics (Kalyuga and Plass, 2025). As discussed in the previous section, it could be the case that children are just more familiar with solving arithmetical tasks that are text-based, and that would mean that the intrinsic load of the text-based task is lower due to children’s familiarity with the task type, and/or that they have more developed cognitive schemas to extract the arithmetic-relevant information in a text-based task than in one with visual aid. This would suggest the conclusion that if two tasks – A and B – have the same content, and the participants are more familiar with A than B, then A has the lower intrinsic load. Thus, the result shows the need for explication within cognitive load theory of how we should understand the difference between the intrinsic load with the same content ( a+b ).

These problems are not only fundamental for scientific purposes when we want to understand phenomena such as cognitive load, but they have practical implications as well. If the theory prescribes reducing the extraneous load, it is of critical importance to identify the extraneous load. For a task such as a+b , where we want the child to focus on the arithmetic, should we use a text-based task over a visual aid task if the visual aid adds extraneous load, *or* should we use a visual aid task if the visual-aided task has higher intrinsic load even if the content is the same? The inconsistency identified here makes the distinction moot on guiding practice, which is problematic and calls for further research and perhaps increased theoretical clarity.

## 5 Limitations

Besides possible confounders that were not handled by the design, as mentioned in Section 4.1, there are additional limitations that affect the interpretation of the data. One limitation is that we only matched the arithmetic difficulty between the different task types, whereas a stronger design would have the exact same questions, but randomized, with visual aid or not. We also did not utilize any eye tracking or distraction conditions, which could have been used as a manipulation check for the perceptual load.

Moreover, the study only included 9-year-olds and additive situations with numbers between 1 and 30. Thus, the claims regarding cognitive load increase due to visual aids should not be generalized to other forms of mathematics and to older children, such as high school students. Regarding the functional brain imaging, we only measured over the frontal cortex, and fNIRS is not able to measure subcortical areas. This did not affect the ability to evaluate the hypotheses of the study, but it limits our ability to discuss the involvement of other brain areas. There are dynamic parts of cognitive load that are possible to investigate with functional brain imaging, such as temporal connectivity, which were not addressed in this study. To handle the difference in response time between participants, we used peak oxy-Hb, but this is based on each experimental condition and does not capture the variation within each experimental condition.

Due to the wavelength combination (780 nm, 850 nm), oxy-Hb is typically estimated with higher sensitivity than deoxy-Hb (Sato et al., 2013; Uludağ et al., 2004). In our data, deoxy-Hb did not show the expected task-evoked decrease (Supplementary Figure 4) and did not differentiate between high load and low load (Supplementary Table 5), limiting the interpretability of deoxy-Hb. We therefore used oxy-Hb as the primary outcome and analyzed deoxy-Hb in parallel as a quality check to ensure that oxy-Hb effects were not accompanied by same-direction deoxy-Hb effects (Supplementary Tables 5, 6). Nevertheless, the lack of a canonical deoxy-Hb response warrants cautious interpretation of the fNIRS findings.

The Homer3 GLM estimated the HRF using Gaussian basis functions, which allow for non-canonical HRF shapes. However, large variability in response times across children may still have introduced temporal jitter and overlap with adjacent trials within the regression window. While a deconvolutional/finite impulse response GLM may better capture timing variability, it would substantially increase model complexity and require trial structure/timing that supports stable estimation. Future studies should collect and incorporate individual response timing to facilitate deconvolutional approaches.

## 6 Conclusion

Contrary to our hypothesis, visual aids during text-based arithmetic tasks increased the cognitive load compared to only text-based tasks for 9-year-olds. Even though the arithmetic content of the task was matched, children made comparatively more errors and answered more slowly on tasks with visual aids. With the use of the increased working memory paradigm, we were able to experimentally isolate the left and right frontal polar areas (FPA) (i.e., the anterior part of the prefrontal cortex) as being involved when the working memory increased during arithmetic tasks. We could also provide anecdotal to substantial evidence for an increase in the right frontal polar area during the visual aid task compared to only text-based tasks. The addition of irrelevant visual information to a text-based task did not affect the score but increased the time to answer.

## Data Availability

The datasets presented in this study can be found in online repositories. The names of the repository/repositories and accession number(s) can be found at: https://osf.io/mprcy/files/osfstorage.

## Footnotes

1 There are some versions of the Cognitive Load Theory that defines intrinsic load as: “[T]he load that is relevant to learning as schema acquisition (Kalyuga and Plass, 2025). This definition would make Cognitive Load Theory mute on questions regarding performance, thus irrelevant for the purpose of this paper.

2 It should be remembered that effects are empirical regularities/phenomena not tied or owned by any particular theory, and should be explained by every theory having that phenomena within its boundary conditions. For example, the Stroop effect, i.e., individuals are slower to state the ink color of the word RED printed in blue ink compared to the word RED in red ink. The Stroop effect is a pattern in data or a phenomenon in the world that every theory about attention or processing need to explain (be it the Resource Theory of Attention or the Tectonic Theory of attention or something else). The same with the different “cognitive load theory effects”, they cannot be deduced from any current version of the cognitive load theory, and as has been pointed out by its proponents, they are added extra theoretical postulates due to failure of getting the expected results in previous studies (Sweller, 2023). One can of course put in the empirical phenomena (the effects) in to the theory as an axiom, but then one still fails to explain them, since now it is only a part of the axioms of the theory. In the same way as Euclidean geometry does not explain why two parallel lines never meet, it only states it as an axiom.

3 Most brain image studies that investigate cognitive load have not been conducted take out in an educational setting, but rather in a medical, brain-computer interaction or driving science setting, and do not conceptualize cognitive load the same way Cognitive Load Theory researchers do. For example, in a review by Ayres et al. (2021) of physiological measure of intrinsic cognitive load, they found 13 neuroimaging studies. Here only one study. Vanneste et al. (2021) used the conceptualization according to the Cognitive Load Theory.
