# Supplementary material for: Visualizations during arithmetic tasks hamper performance and increase cognitive load in 9-year-olds: an fNIRS study
Source: Front Psychol. 2026 Feb 11;17:1722948. doi: 10.3389/fpsyg.2026.1722948 (PMC12932464; doi:10.3389/fpsyg.2026.1722948)
Supplement: Supplementary file 1 [file Supplementary_file_1.docx]

Supplementary Material

**Visualizations during arithmetic tasks hamper performance and increase cognitive load in 9-year-olds, an fNIRS study**

Simon Skau^1*^, Jimmy Karlsson^1^, Jakob Åsberg Johnels^2^, Ola Helenius^3^

^1^ *Department of Mathematics and Computer Science, Karlstad University, Karlstad, Sweden*

^2^ Department of Education and Special Education, Faculty of Education, University of Gothenburg, Gothenburg, Sweden

^3^ Department of Pedagogical, Curricular and Professional Studies, Faculty of Education, University of Gothenburg, Gothenburg, Sweden

*** Correspondence:**Simon Skau
simon.skau@kau.se

**Preliminary analysis**

Descriptive statistics of the Math task is summarized in Supplementary Table 1.

**Supplementary Table 1.** Descriptive statistic of Math task.

|  | **Score** | | **Time to answer (seconds)** | |
| --- | --- | --- | --- | --- |
|  | Mean (SD) | Min Max | Mean (SD) | Min Max |
| Low Load | 24.0 (4.5) | (10 30) | 13.6 (4.1) | (6 23.9) |
| High Load | 17.7 (5.8) | (3 29) | 16.0 (3.7) | (9.3 24.0) |
| Visual Low Load | 7.2 (1.9) | (3 10) | 14.8 (4.3) | (6.2 24.2) |
| Visual High Load | 5.5 (2.4) | (0 10) | 17.5 (4.2) | (9.0 26.0) |
| Text Low Load | 8.4 (1.5) | (3 10) | 12.5 (4.4) | (5.8 24.5) |
| Text High Load | 6.1 (2.1) | (1 10) | 14.5 (3.8) | (7.4 23.3) |
| Irrelevant Low Load | 8.3 (1.7) | (3 10) | 13.7 (4.3) | (6.8 25.4) |
| Irrelevant High Load | 6.0 (1.9) | (1 10) | 15.2 (3.8) | (7.7 25.4) |

The first preliminary analysis, to assess the difficulty of the tasks, was the unidimensional Rasch analysis. The β values for the 60 tasks are presented in Supplementary **Figure 1A)**. The results of the Rasch analysis indicate that the Low Load task is not particularly difficult for these participants, as most of the β values are negative, with the mean and standard deviation (SD) of -1.29 (±1.32), -2.43 (±1.45) and -2.26 (±1.40) for Visual aid, Text-based and Irrelevant task respectively. In comparison, the High Load task is more challenging than the Low Load task; with a mean -0.06 (±0.80) for the Visual aid, -0.69 (±1.60) for the Text-based and -0.66 (±1.63) for the Irrelevant task. The test would not be considered hard as the overall mean and SD are -1.23 (±1.59) (for all values see data file at [*https://osf.io/mprcy/files/osfstorage*](https://osf.io/mprcy/files/osfstorage)).

The second preliminary analysis is to evaluate if there are any fatigability effect, i.e. are the tasks harder or does the time to answer increase the longer the test goes. Supplementary **Figure 1**B show β value for each task (in blue) and mean time to answer in seconds (in orange) over the 60 tasks. Neither the correlations between *Task Order* and *β value* (τ =-0.167, BF_10_ = 0.96 or BF_01_ =1.03) nor between and *Task Order* and *Mean* *Time to Answer* (τ =-0.141, BF_10_ = 0.58 or BF_01_ =1.71) give us reason to believe that there is any fatigability effect; if anything, it gives anecdotal evidence for the opposite.

| 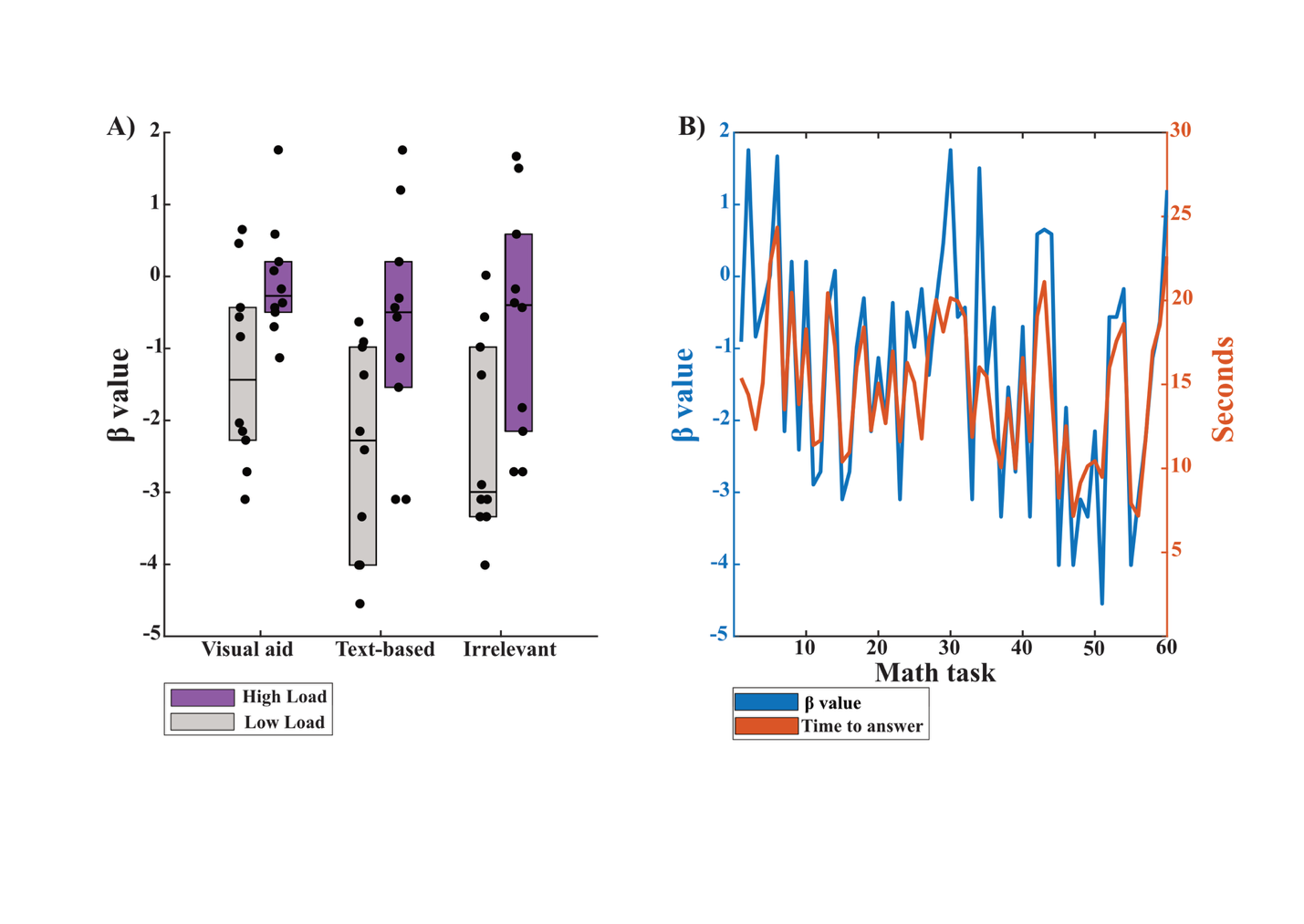 |
| --- |
| **Supplementary Figure 1. A)** β values from the Rasch analysis for all the Math tasks. **B)** β values (in blue and left y-axis) and time to answer (orange and right y-axis) plotted for each task in order (x-axis). |

Since the correlations are inconclusive, we compared the performance of the first 10 task with the performance of the last 10 task with a paired t-test for accuracy and time to answer. The analysis shows that the participants made more errors in the beginning (43% errors) compared to the last 10 (29% error) with a BF_10_ > 1000, and they are slower in the beginning with of an average of 16.97 seconds to answer compared to 14.65 seconds to answer with a BF_10_ > 1000).

Thirdly, as summarized in Supplementary Table 2, the correlation between time to answer and score tended to be stronger within each task condition. This was the case for Visual Low (τ = -0.55), Visual High (τ = -0.48), Text Low (τ = -0.54), Irrelevant Low (τ = -0.57) and Irrelevant High (τ = -0.49). It is only Text High time to answer that had the strongest correlation with Text Low score (τ = -0.51) compared to Text High score (τ = -0.45). For the aggregated constructs (Low and High Load), Time Low Load have a stronger correlation with Score Low Load compared to Score High Load (τ = -0.61 compared to tau = -0.46), but Time High Load have a stronger correlation with Score Low Load compared to Score High Load (τ = -0.53 compared to τ = -0.51).

**Supplementary Table 2.** Correlation between score and time to answer.

|  | **Score** | | | | | | | |
| --- | --- | --- | --- | --- | --- | --- | --- | --- |
| **Time** | **Low** | **High** | **Visual Low** | **Visual High** | **Text Low** | **Text High** | **Irrelevant Low** | **Irrelevant High** |
| **Low** | -0.617 | -0.466 | -0.564 | -0.407 | -0.548 | -0.448 | -0.522 | -0.407 |
| **High** | -0.536 | -0.51 | -0.462 | -0.454 | -0.491 | -0.458 | -0.512 | -0.438 |
| **Visual Low** | -0.574 | -0.458 | -0.552 | -0.428 | -0.488 | -0.414 | -0.482 | -0.381 |
| **Visual High** | -0.495 | -0.495 | -0.449 | -0.485 | -0.402 | -0.419 | -0.475 | -0.421 |
| **Text Low** | -0.582 | -0.439 | -0.51 | -0.349 | -0.544 | -0.446 | -0.495 | -0.389 |
| **Text High** | -0.498 | -0.457 | -0.411 | -0.348 | -0.513 | -0.455 | -0.45 | -0.406 |
| **Irrelevent Low** | -0.639 | -0.433 | -0.552 | -0.352 | -0.556 | -0.447 | -0.572 | -0.395 |
| **Irrelevent High** | -0.496 | -0.49 | -0.429 | -0.337 | -0.467 | -0.494 | -0.469 | -0.492 |

***Bayesian Kendall's Tau Correlations***

As the correlation between the score and time to answer are indicators of the same performance, we combined the two by z-transforming the results for scores and time to answer (as described in 2.6) and took the mean of the two as the variable of interest. With this new variable, we could do the fourth preliminary analysis, to compare it to the other three mathematical tasks. The correlation with the other mathematical task gave extreme evidence for a medium to strong correlation with the weakest correlation for Irrelevant Low Load and AS r = 0.44 and the strongest for overall High Load and BANUCA with AS r = 0.69 (see Supplementary Table 3 for all correlations).

**Supplementary Table 4.** Correlation between with the mathematical tasks.

|  |  | **BANUCA** | **AMR** | **AS** |
| --- | --- | --- | --- | --- |
| **Low Load** | r | 0.633 | 0.646 | 0.511 |
|  | BF₁₀ | >1000 | >1000 | >1000 |
| **High Load** | r | 0.697 | 0.677 | 0.568 |
|  | BF₁₀ | >1000 | >1000 | >1000 |
| **Visual Low Load** | r | 0.667 | 0.61 | 0.504 |
|  | BF₁₀ | >1000 | >1000 | >1000 |
| **Visual High Load** | r | 0.614 | 0.571 | 0.502 |
|  | BF₁₀ | >1000 | >1000 | >1000 |
| **Text Low Load** | r | 0.549 | 0.618 | 0.479 |
|  | BF₁₀ | >1000 | >1000 | >1000 |
| **Text High Load** | r | 0.62 | 0.668 | 0.569 |
|  | BF₁₀ | >1000 | >1000 | >1000 |
| **Irrelevant Low Load** | r | 0.54 | 0.574 | 0.44 |
|  | BF₁₀ | >1000 | >1000 | >1000 |
| **Irrelevant High Load** | r | 0.684 | 0.626 | 0.476 |
|  | BF₁₀ | >1000 | >1000 | >1000 |

Together, these preliminary analyses give us reason believe that, the tasks were not too hard and there was no fatigability, thus giving us reason to trust the data for the whole test session. Additionally, the score and time to answer reflected similar performance and the Math task had a medium to strong correlation to the other mathematical tasks (AS, BANUCA and ARM) giving us additional reason to trust that the Math task measures relevant mathematical ability.

**Supplementary information to the main analysis**

First, we have the statistics that make up the behavioral analysis summarized in Supplementary Table 4.

**Supplementary Table 4.** Test statistics for paired t-tests.

|  |  |  |  | **Score** | | | **Time to answer (seconds)** | |
| --- | --- | --- | --- | --- | --- | --- | --- | --- |
|  |  |  | **BF₁₀** | | **W** | **Effect*** | **BF₁₀** | **Effect*** |
| Low Load | vs. | High Load | >1000 | | 3218 | 2.13 | >1000 | -0.89 |
| Visual Low Load | vs. | Visual High Load | >1000 | | 2299 | 1.08 | >1000 | -0.82 |
| Text Low Load | vs. | Text High Load | >1000 | | 2548 | 1.79 | >1000 | -0.58 |
| Irrelevant Low Load | vs. | Irrelevant High Load | >1000 | | 2709 | 1.51 | 131.05 | -0.42 |
| Visual Low Load | vs. | Text Low Load | >1000 | | 154 | -0.93 | >1000 | 0.76 |
| Visual Low Load | vs. | Irrelevant Low Load | >1000 | | 215 | -0.82 | 35.11 | 0.38 |
| Text Low Load | vs. | Irrelevant Low Load | 0.142 | | 563 | 0.05 | 196.48 | -0.43 |
| Visual High Load | vs. | Text High Load | 4.313 | | 599 | -0.30 | >1000 | 0.90 |
| Visual High Load | vs. | Irrelevant High Load | 1.467 | | 632 | -0.24 | >1000 | 0.64 |
| Text High Load | vs. | Irrelevant High Load | 0.153 | | 970 | -0.06 | 0.913 | -0.22 |

*Median effect; degree of freedom 80 for all

The regions of interest is based on the paired t-test. In Supplementary Table 5 all statics are presented both for oxy-Hb and deoxy-Hb. They are presented together in order to show that that the results from deoxy-Hb does not show similar result as oxy-Hb. And since oxy-Hb converges with the behavioral data this gives us further evidence not to trust the deoxy-Hb data, besides the argument made in the main text. The channel location is presentenced in Supplementary Figure 3. The deoxy-b curves are visualized in Supplementary Figure 4.

**Supplementary Table 5.** Oxy-Hb and deoxy-Hb High Load vs Low Load (paired t-test).

|  | **Oxy-Hb** | | | **Deoxy-Hb** | | |
| --- | --- | --- | --- | --- | --- | --- |
| **Channel** | **df** | **t values** | **BF₁₀** | **df** | **t values** | **BF₁₀** |
| 1 | 79 | 4.72 | 1698.29 | 79 | -0.45 | 0.13 |
| 2 | 77 | 3.19 | 12.85 | 77 | -0.54 | 0.14 |
| 3 | 79 | 4.73 | 1777.59 | 79 | 0.77 | 0.16 |
| 4 | 77 | 2.47 | 2.146 | 77 | -1.39 | 0.31 |
| 5 | 71 | 2.21 | 1.226 | 71 | 0.55 | 0.14 |
| 6 | 73 | 2.77 | 4.241 | 73 | -1.18 | 0.24 |
| 7 | 79 | 2.90 | 6.05 | 79 | 0.67 | 0.15 |
| 8 | 79 | 2.26 | 1.36 | 79 | 1.22 | 0.25 |
| 9 | 78 | 3.19 | 12.71 | 78 | 1.94 | 0.73 |
| 10 | 79 | 1.85 | 0.63 | 79 | -1.11 | 0.22 |
| 11 | 77 | 1.52 | 0.37 | 77 | 1.03 | 0.20 |
| 12 | 76 | 2.42 | 1.93 | 76 | -1.20 | 0.24 |
| 13 | 78 | 0.71 | 0.15 | 78 | 2.71 | 3.71 |
| 14 | 65 | 2.77 | 4.23 | 65 | -1.72 | 0.50 |
| 15 | 74 | 2.48 | 2.17 | 74 | -1.60 | 0.42 |
| 16 | 71 | -0.21 | 0.12 | 71 | -1.16 | 0.23 |
| 17 | 72 | 0.77 | 0.16 | 72 | 0.56 | 0.14 |
| 18 | 60 | 2.15 | 1.08 | 60 | -0.20 | 0.12 |
| 19 | 49 | -0.44 | 0.13 | 49 | 0.53 | 0.14 |
| 20 | 72 | 1.35 | 0.29 | 72 | 0.71 | 0.15 |
| 21 | 71 | 0.71 | 0.15 | 71 | 1.78 | 0.55 |
| 22 | 63 | 1.35 | 0.295 | 63 | -1.19 | 0.24 |
| 23 | 63 | 1.79 | 0.56 | 63 | -1.26 | 0.26 |
| 24 | 67 | 0.08 | 0.12 | 67 | 0.12 | 0.12 |
| 25 | 57 | 0.48 | 0.13 | 57 | 0.61 | 0.14 |
| 26 | 74 | 4.19 | 258.53 | 74 | -0.21 | 0.12 |
| 27 | 77 | 1.29 | 0.27 | 77 | 1.25 | 0.26 |
| 28 | 75 | 1.36 | 0.29 | 75 | -1.14 | 0.23 |
| 29 | 76 | 1.88 | 0.66 | 76 | 3.01 | 7.82 |
| 30 | 71 | 0.54 | 0.14 | 71 | -1.05 | 0.21 |
| 31 | 72 | 1.36 | 0.30 | 72 | 0.67 | 0.15 |
| 32 | 68 | 0.60 | 0.14 | 68 | -1.85 | 0.62 |
| 33 | 76 | 1.58 | 0.41 | 76 | 0.35 | 0.13 |
| 34 | 76 | 0.94 | 0.18 | 76 | 0.30 | 0.12 |
| 35 | 77 | 2.08 | 0.95 | 77 | -1.41 | 0.32 |
| 36 | 67 | 0.45 | 0.13 | 67 | 0.55 | 0.14 |
| 37 | 77 | 0.40 | 0.13 | 77 | -1.77 | 0.55 |
| 38 | 72 | 0.93 | 0.18 | 72 | -0.43 | 0.13 |
| 39 | 73 | 1.55 | 0.39 | 73 | -1.34 | 0.29 |
| 40 | 63 | -0.38 | 0.13 | 63 | -0.85 | 0.17 |
| 41 | 70 | 0.48 | 0.13 | 70 | 0.14 | 0.12 |
| 42 | 65 | -0.73 | 0.16 | 65 | -0.11 | 0.12 |
| 43 | 62 | 1.05 | 0.20 | 62 | 1.47 | 0.34 |
| 44 | 52 | 1.59 | 0.41 | 52 | 0.53 | 0.14 |

Visualization of the secondary analysis is shown in Supplementary Figure 2.

| 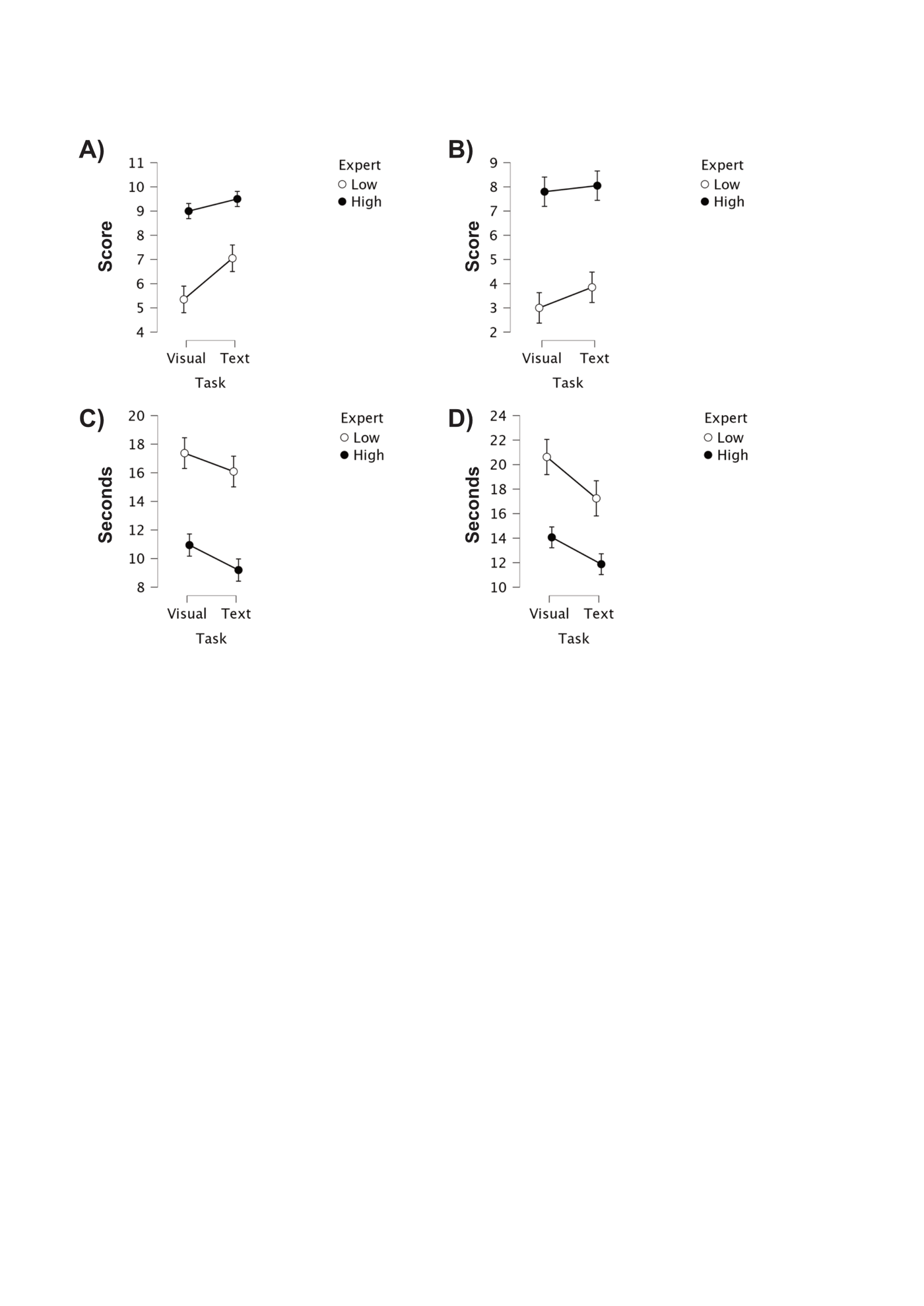 |
| --- |
| **Supplementary Figure 2. A)** Score Low load, **B)** Score High load, **C)** Low load time to answer and **D)** High load time to answer. |

| 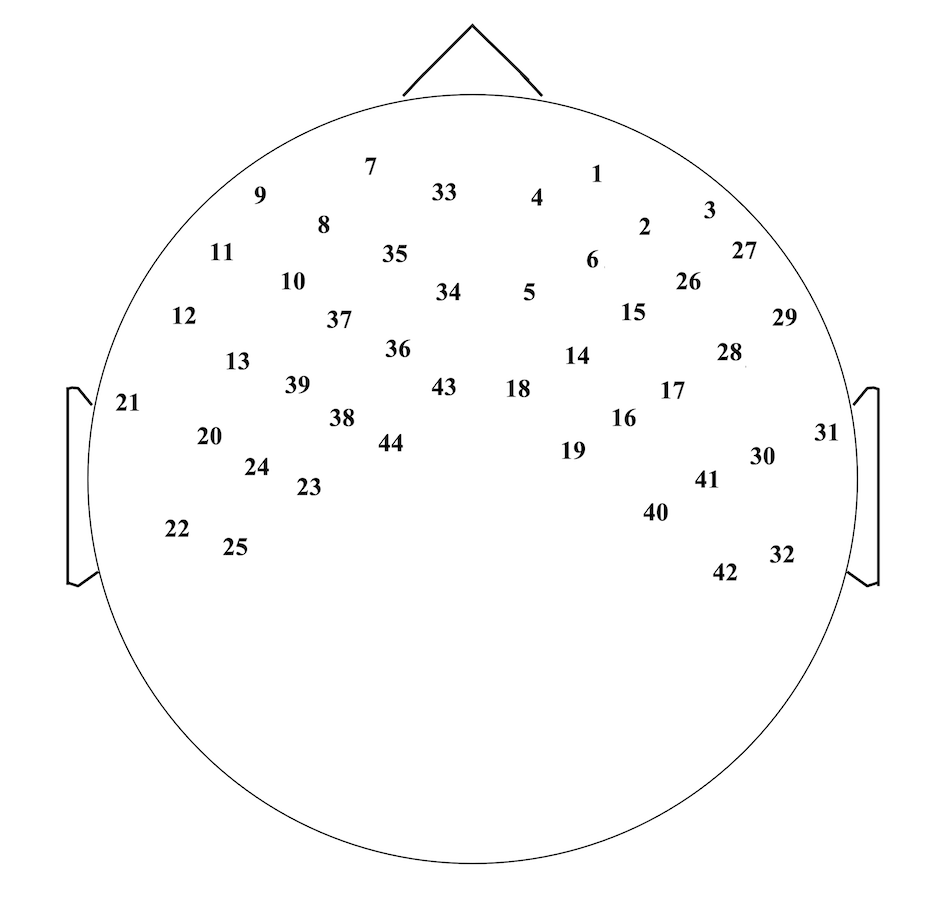 |
| --- |
| **Supplementary Figure 3.** Channel layout of the fNIRS channels. |

| 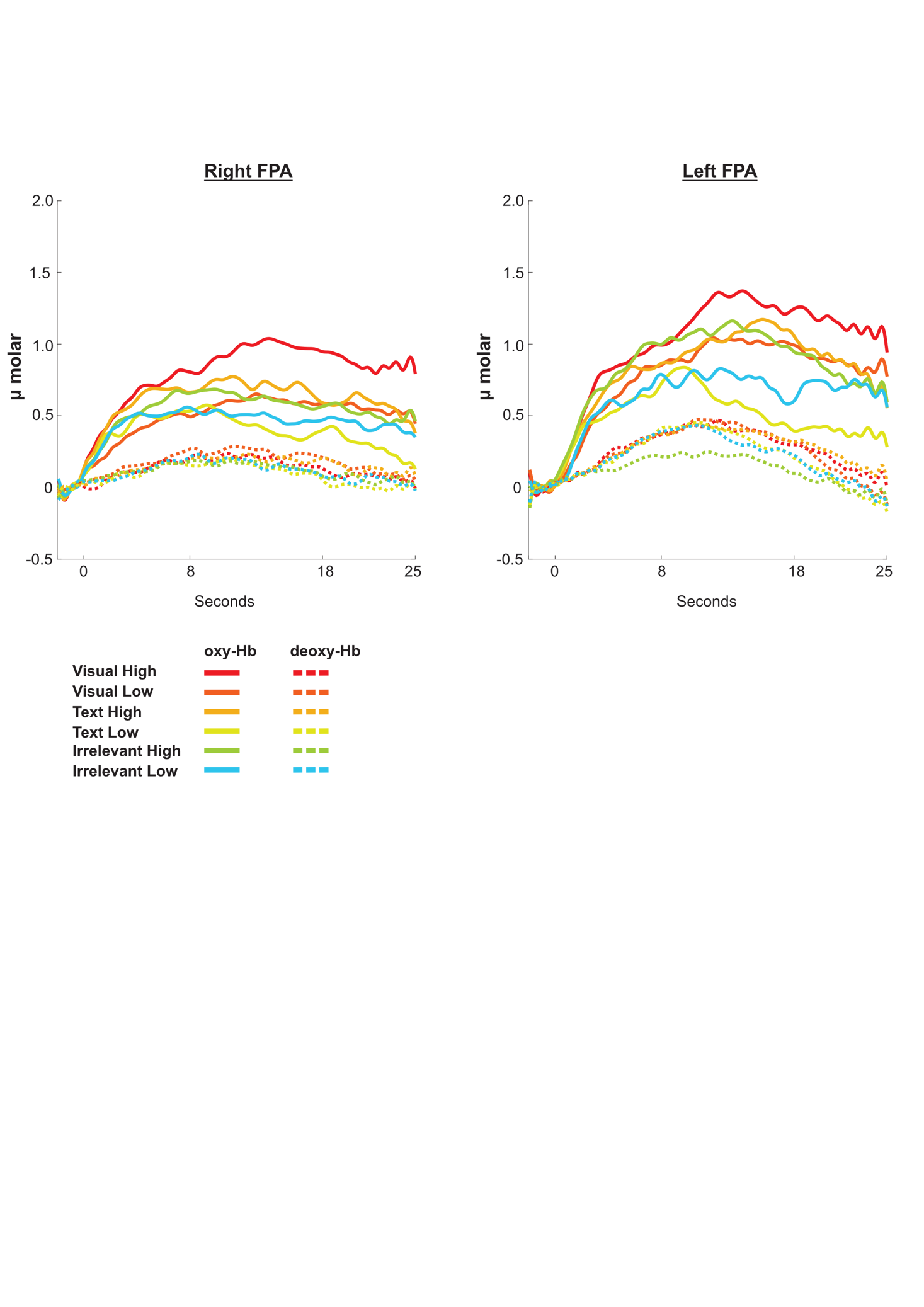 |
| --- |
| **Supplementary Figure 4.** Oxygenated and deoxygenated curves for right and left frontal polar area during the different conditions. |

**Supplementary Table 6.** Deoxy-Hb contrasts (paired t-test).

|  |  |  | **Right FPA** | | **Left FPA** | |
| --- | --- | --- | --- | --- | --- | --- |
|  |  |  | **BF₁₀** | **t value** | **BF₁₀** | **t value** |
| **High Load** | **vs.** | **Low Load** | 0.1241 | 0.1210 | 0.2955 | 1.3522 |
| **Visual High Load** | **vs.** | **Visual Low Load** | 0.3103 | -1.3900 | 0.1964 | 0.9845 |
| **Text High Load** | **vs.** | **Text Low Load** | 0.2638 | 1.2608 | 0.3124 | 1.3951 |
| **Irrelevant High Load** | **vs.** | **Irrelevant Low Load** | 0.1709 | 0.8243 | 0.1789 | -0.8798 |
| **Visual Low Load** | **vs.** | **Text Low Load** | 2.8658 | 2.6025 | 0.1298 | 0.3272 |
| **Visual High and Low Load** | **vs.** | **Text High and Low Load** | 0.6426 | 1.8674 | 0.1254 | 0.1908 |
